# Underwater Computer Vision for Ecologists: A Framework for Curating Image Training Datasets for Object Detection

**DOI:** 10.3390/s26185869

**Published:** 2026-09-16

**Authors:** Talen Rimmer, Colin Bates, Declan McIntosh, Tom Zhang, Alexandra Branzan Albu, Francis Juanes

**Affiliations:** 1 Department of Biology, University of Victoria, Victoria, BC V8P 5C2, Canada; tomzhang1257@uvic.ca (T.Z.); juanes@uvic.ca (F.J.); 2 Cascadia Seaweed, Sidney, BC V8L 3A4, Canada; cbates@cascadiaseaweed.com; 3 Department of Electrical and Computer Engineering, University of Victoria, Victoria, BC V8P 5C2, Canada; declanmcintosh@uvic.ca (D.M.); aalbu@uvic.ca (A.B.A.)

**Keywords:** ecology, biodiversity, object detection, underwater imagery, image annotation, annotation methods

## Abstract

As marine ecosystems experience accelerating change, there is an urgent need for efficient and scalable biodiversity monitoring tools. We present a 10-step framework for integrating computer vision (CV) tools into long-term underwater biodiversity monitoring, using a case study from coastal British Columbia. Over 9000 h of unbaited remote underwater video footage were collected from two kelp farms and reference sites between March 2022 and June 2023. The framework includes steps for creating an annotated training dataset using unsupervised and supervised CV tools, culminating in the training and validation of a YOLOv8 object detection model. This process produced over 241,000 annotations across 54 pseudo-taxonomic categories (representing both taxa and visually similar groups of fauna), with a focus on fish and gelatinous zooplankton groups. The final model achieved an overall F1 (2 × precision × recall/(precision + recall)) of 0.74 and a mean average precision at 0.5 intersection over union (mAP50) of 0.78. The model had the highest performance on fine-resolution taxa such as *Phanerodon vacca* (F1 = 0.88) and *Aurelia labiata* (F1 = 0.90), and lowest performance on pseudo-taxa with limited visual distinctiveness such as Actinopterygii (F1 = 0.60) and Cnidaria (F1 = 0.60). A re-training experiment using annotation thresholds between 25 training images to full dataset availability (~200–24,000 images per group) found that model performance was positively correlated with annotation effort, with F1 averaging 0.84 and mAP50 averaging 0.91 at the maximum training dataset size. Our results suggest that the model is most effective for abundant and visually distinctive taxa, while performance declines for groups with coarse taxonomic resolution. We recommend optimizing annotation effort by targeting genus- or species-level taxa, having at least one broad-level group to capture order-level abundances, and supplementing annotations of rare groups with common but morphologically similar groups, which may further improve model performance.

## 1. Introduction

As global marine ecosystems undergo rapid and complex transformations, ecologists require monitoring tools that are not only scalable and cost-effective, but can also deliver taxonomic insights over long-term temporal and spatial scales. Conventional methods such as diver surveys, Remote Operated Vehicle (ROV) deployments, and Baited Remote Underwater Video (BRUV) have provided essential baselines for monitoring but are often constrained by the cost and time to review collected videos or images, and limited temporal coverage (e.g., [1,2]).

In recent years, image-based methods such as RUVs have gained traction for their ability to capture continuous, long-term data in a non-invasive manner (e.g., [3,4,5,6]). This method involves an unbaited underwater camera deployed for long periods of time at a site, minimizing biases that can impede accurate community data such as ‘carnivore attraction’ in BRUVs [4,7] and an ‘observer effect’ in dive [8,9] and ROV [10] surveys. Recent advancements in camera equipment allow for RUVs to be programmed to turn off and on at regular intervals, letting researchers better select the period of time they want to capture (e.g., [11]). However, this method is also highly time- and cost-intensive for annotation, as collected videos typically must be painstakingly reviewed by relevant experts to produce community-level diversity results (e.g., [6]).

Computer vision (CV) techniques, which use computer algorithms to assess images and videos, present a promising approach for automating the identification, counting, and assessment of taxa in underwater imagery. However, their application in marine systems is relatively limited, especially in complex underwater environments [12], due in part to several fundamental challenges. Firstly, the successful implementation of computer vision tools requires well-annotated datasets, specialized data pipelines, and meticulous attention to the model’s application, especially when evaluating multiple taxa (e.g., [13,14,15]). Moreover, the use of CV in underwater ecology is hindered by the unpredictability of animal occurrences in camera footage, which frequently result in imbalanced datasets and ultimately fewer annotations for rare and cryptic taxa, leading to poor model generalization (e.g., [14,16]). Lastly, there is no consensus on the minimum number of annotations needed for object detection models to accurately identify and count various underwater taxa. Limited evidence suggests that achieving high model performance (e.g., >90% precision) on benthic epifauna may require over one thousand annotations [17], whereas fish may require up to several thousand annotations per species [13,18], but this can vary widely across the environment, species, and camera setup.

This study examines the above challenges through the development and validation of a computer vision framework designed for long-term underwater biodiversity monitoring. Specifically, we present a case study from coastal British Columbia, where CV techniques were applied to assess epipelagic macrofaunal diversity through long-term RUV deployments. Conducted from March 2022 to June 2023, the study collected over 9000 h of underwater video from two seaweed farms and nearby reference sites. The primary aim of the initial project [19] was to assess the use of coastal kelp farms as habitat by salmonids and their prey over an 18-month period, using a supervised object detection algorithm (YOLOv8) to assist in automated image-based identification.

Here, we provide a practical framework for using computer vision in long-term underwater monitoring. We begin by detailing the data collection and validation methods used to train the YOLOv8 object detection model, followed by an experiment designed to evaluate annotation effort requirements, resulting in an integrated ecological annotation framework using well-known object detection and image classification algorithms. We then discuss tools for validating computer vision models in ecological monitoring and assess the strengths and limitations of our approach, with a focus on how we addressed the annotation-related challenges presented above. Finally, we propose future directions for refining these methods, and explore how ecologists can integrate CV tools into existing monitoring programs.

## 2. Methods

### 2.1. Video Data Collection

**Figure 1:**
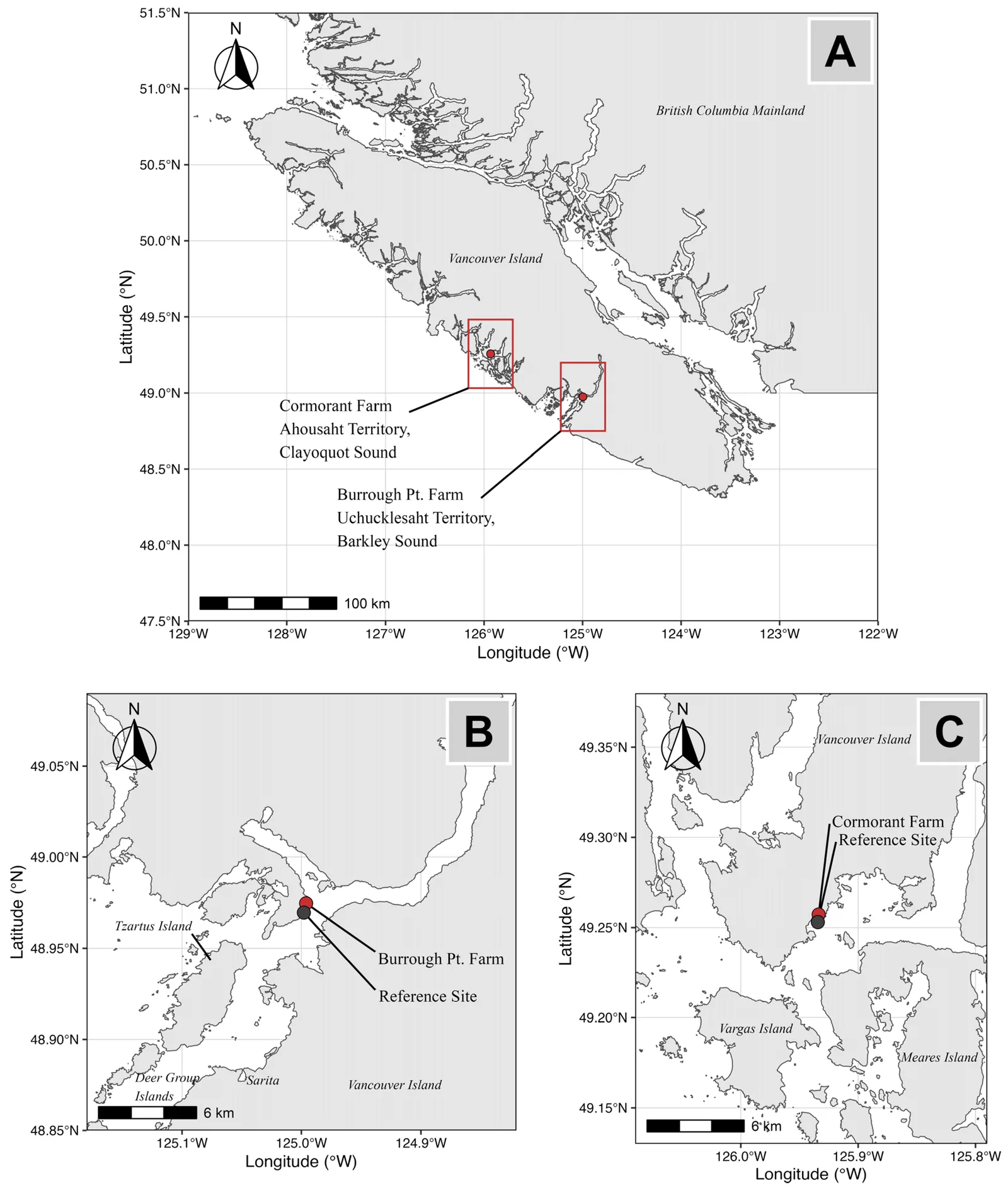
Location of Cascadia seaweed farms near Vancouver Island (**A**), and the location of the farms and reference sites within Barkley (**B**) and Clayoquot (**C**) Sounds.

Video data were supplied by a project that monitored fish diversity and abundance over an 18-month period on two seaweed farms in coastal British Columbia [19]. The farms were located at Burrough Point in Barkley Sound (Uchucklesaht Territory; 49°15′24.95″ N, 125°55′28.05″ W) and Cormorant Point in Clayoquot Sound (Ahousaht Territory; 48°58′29.21″ N, 124°59′46.15″ W) (Figure 1). The reference site for Burrough Point was located ~750 m southwest of the farm (48°58′05.98″ N, 124°00′01.27″ W), and the reference site for the Cormorant farm was located ~600 m to the north (49°15′45.57″ N, 125°55′19.77″ W).

**Figure 2:**
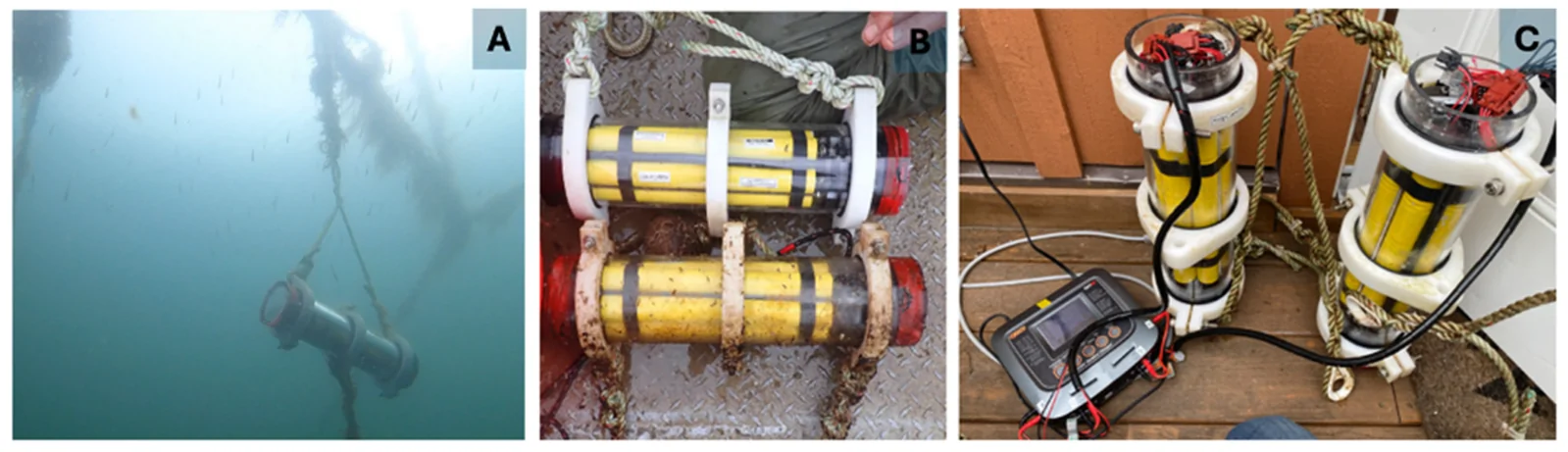
FishCams were built by Cascadia Seaweed (**B**,**C**) and deployed at two seaweed farms in coastal British Columbia (**A**). Picture credit: Colin Bates.

Video data were collected using programmable unbaited underwater video cameras that were designed and presented in Mouy et al. (2020) [11] and modified for the purposes of this project (henceforth referred to as ‘FishCams’, Figure 2). The cameras consisted of a Raspberry Pi Zero W single-board computer (Raspberry Pi Foundation, Cambridge, UK), a WittyPi Mini 3 power management board (UUGear, Prague, Czech Republic), and a 12.3 megapixel camera with a 6 mm wide-angle lens with a 63° field angle. Videos were recorded as H.264 video streams with a resolution of 1600 × 1200 pixels, auto exposure, and auto white balance. Each camera was encased in commercially available 4″ waterproof housing (Blue Robotics, Torrance, CA, USA). The complete technical specifications of the modified FishCams, including detail of the materials and costs required in their production, can be found in Bates and Pedde [20].

**Figure 3:**
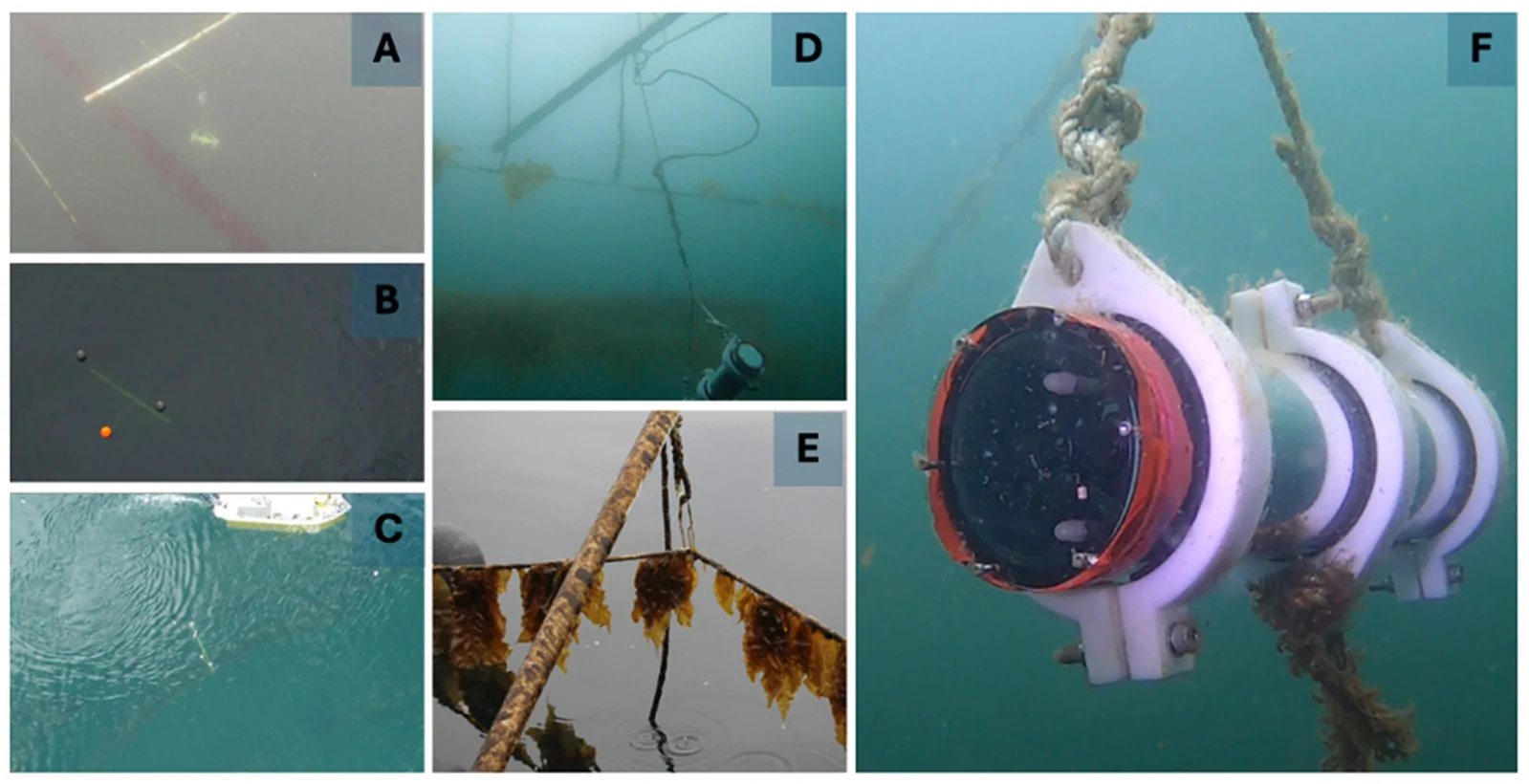
Overhead view of deployment configuration (**A**–**C**) and underwater mounting of FishCams (**D**–**F**) at each seaweed farm (**A**) and adjacent reference site (**B**). Picture credit: Colin Bates.

FishCams were deployed and retrieved monthly from March 2022 to June 2023. Cameras were arranged such that three FishCams were inside each farm, and three at each adjacent reference site (12 cameras total; Figure 3). Cameras at the farm sites were attached via mooring rope directly to growing lines using a spreader bar system (Figure 2A). At the reference sites, cameras were deployed at 6 m depth, each hung from a dedicated mooring supported by a Scotchman buoy (Cascadia Seaweed, Sidney, BC, Canada) to mimic the depth of cameras at kelp farms. Pendulum counterweights (4.54 kg cannonball on a 30 cm rope tether) were attached to the bottom of each camera to add stability when surface waters were choppy. The camera lenses at both sites were tilted approximately 10–15 degrees towards the surface as this provided the best lighting, and the camera was allowed to swing freely on the horizontal plane. FishCams recorded approximately six minutes of video footage every hour during the study period, for a total of 9037 h of video data recorded. Cameras were serviced monthly, with de-fouling and camera replacements as required.

### 2.2. Training the Supervised Object Detection Model (YOLOv8)

#### 2.2.1. Conceptualizing a Workflow to Train an Object Detection Model

Given the large volume of video data collected by the FishCams, a thorough plan for training our supervised object detection model (YOLOv8) was required. We understood that training the YOLOv8 model would require substantial input of human-verified images. Thus, we needed to (1) find images that contained animals of interest in the underwater video data, (2) identify them to the lowest possible taxonomic level, and (3) validate those identifications to maintain consistency and accuracy when training the model. These requirements were further complicated by annotation challenges, including unknown detection frequency of animals and a lack of guidelines for training a well-performing model.

We knew that adequately training an object detection model on underwater fauna (i.e., wherein model performance is similar to human annotator accuracy) can require hundreds to several thousand examples of each species (e.g., [13,21]). However, for our taxa of interest (salmonids and their prey) it was not clear how often they would appear in our footage. To address this challenge, we took a precautionary approach and assumed that our taxa of interest would be rare, meaning that a high number of images would need to be manually reviewed to achieve the desired number of annotations for each taxon, and that we would therefore need to prioritize efficiency (minimizing effort per annotation) when annotating the dataset.

Given the high variation in required training data reported in previous similar datasets (e.g., [13,14]), it was not clear how many annotations were needed for each taxon to train an object detection model. To address this challenge, we assumed a minimum baseline of 200 annotations required for each species to be included in the model, as this seemed to be an appropriate balance between what was realistic based on appearance rates of animals in our footage, and the minimum number of annotations that would allow for adequate model performance (as seen in similar datasets like Ditria et al. 2020 [13]). We then conducted an additional experiment that re-trained the model at various quantities of annotations for each group.

Another key challenge was the lack of guidelines on what constitutes an ‘adequately trained’ model for biodiversity monitoring purposes. To address this, we looked to the relevant literature on similar datasets (e.g., [13,14]). Although there is no consensus on acceptable performance thresholds for object detection models that assess ecological data, one option was to aim for similar rates of errors as human annotators [13] or >95% accuracy. Based on a preliminary review of video data that found relatively few animals in our footage, we determined that we would likely be constrained by annotation-effort resources (i.e., time in annotating and quality controlling the dataset) and decided to annotate as many images as possible during the study period.

**Figure 4:**
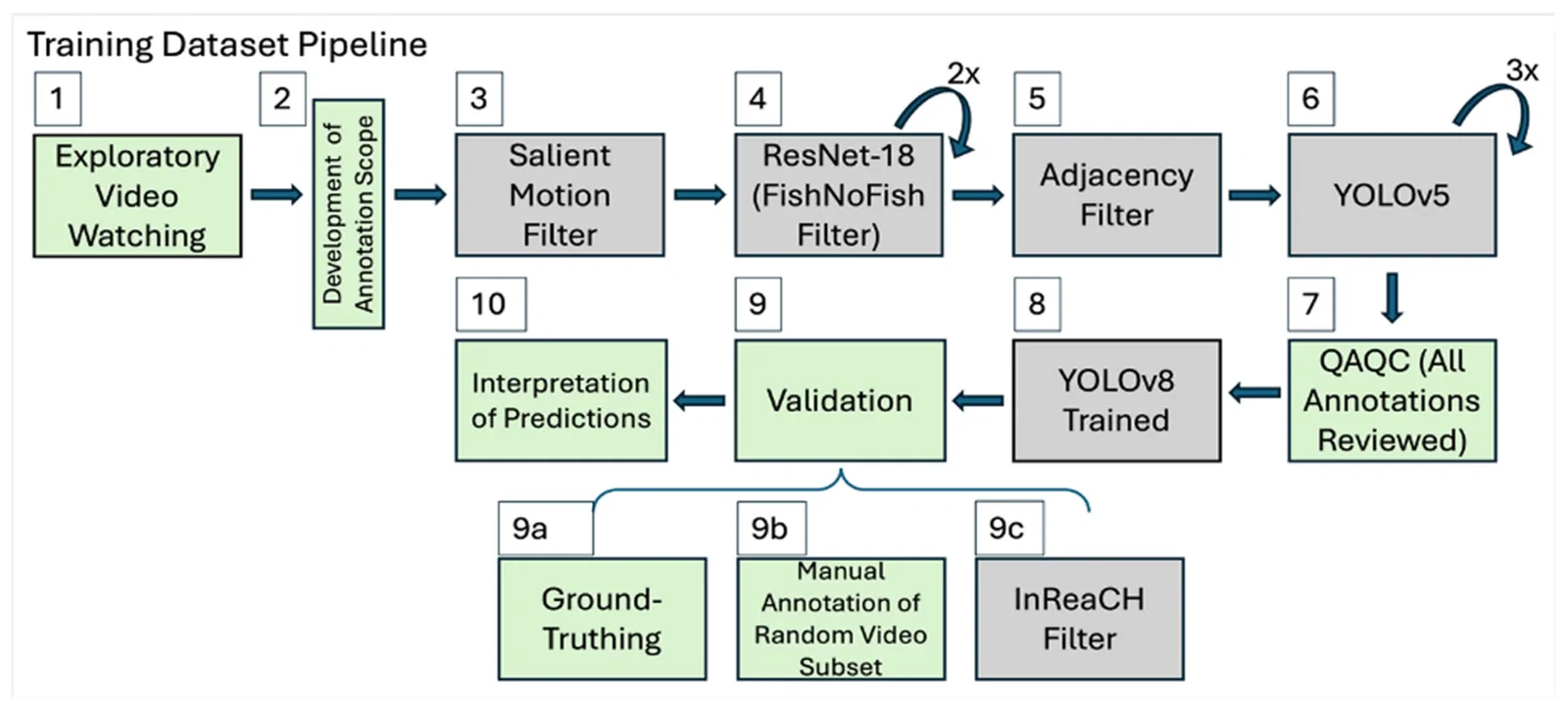
Workflow used to prepare, assess, and validate the annotated training dataset from FishCam videos. Gray and green shading denote steps completed with and without assistance from computer vision tools, respectively. See text (Section 2.2) for a detailed walkthrough of this figure.

With these annotation-related decisions in mind, we developed an iterative 10-step framework to identify and classify marine species from video data (Figure 4). This approach is summarized in the project pipeline flow chart provided in Figure 4 and described in the corresponding text below (Section 2.2.2, Section 2.2.3, Section 2.2.4, Section 2.2.5, Section 2.2.6, Section 2.2.7, Section 2.2.8, Section 2.2.9, Section 2.2.10 and Section 2.2.11). Briefly, the pipeline flows as follows: first, we selected a subset of FishCam video data, collected from March–November 2022, to be used to train the YOLOv8 detector. This date range was chosen because it encompassed approximately one third of the dataset (March 2022–June 2023). Exploratory videos (spanning the first two months of data collection) were first reviewed by annotators to determine video quality, and the appearance rates and movement speed of organisms. Through this process it was noted that a frame rate of approximately one frame per second was appropriate by allowing annotators to accurately identify fast-moving organisms, while necessarily reducing time spent reviewing images. Therefore, we used Python (version 3.X, OpenCV library; [22]) to sequentially extract still images from the raw video at a rate of one frame per second. Additionally, most images contained only open water, so to maximize annotation efficiency we used three sequentially refined screening algorithms to select frames that were more likely to contain objects of interest, prior to training a YOLOv8 object detection model. Each step aimed to iteratively reduce the number of ‘empty’ frames shown to annotators while keeping a high proportion of frames that contained animals of interest. The steps below detail our methodology for annotating the training dataset (Figure 4).

#### 2.2.2. Step 1: Exploratory Video Observation

The initial step in creating our training dataset involved human annotators reviewing raw video data collected for this project (Figure 4). Given that the recording device (FishCams) and the deployment method (direct attachment to seaweed farm infrastructure, Figure 2) were novel, the aim of this step was to assess:

(a) The frequency of animal appearances, particularly salmonids and their prey;
(b) The clarity with which animals could be viewed in the footage;
(c) The common species present;
(d) Any obstructions that could affect future deployments.

Randomly selected videos from a month of data collection (12 cameras) were first reviewed without recording annotations. This step revealed that most videos (>90%) did not contain animals, and those that did were often obscured by high turbidity, fast movement, light attenuation or insufficient camera resolution. This step also confirmed that annotator time would be a limiting factor in gathering the sufficient annotation data needed to train our YOLOv8 model. We therefore developed additional steps to screen out as many ‘empty’ images (i.e., those containing only open water) as possible, while taking care to avoid accidentally removing images of novel animals that might appear in future footage (steps 3–6 in Figure 4).

#### 2.2.3. Step 2: Development of Annotation Methods

To carry out the annotation of organisms contained within individual images, we established a series of annotation rules. The following section details the protocol we used to annotate the training dataset, including key challenges and decisions made.

##### Annotation Rules

**Figure 5:**
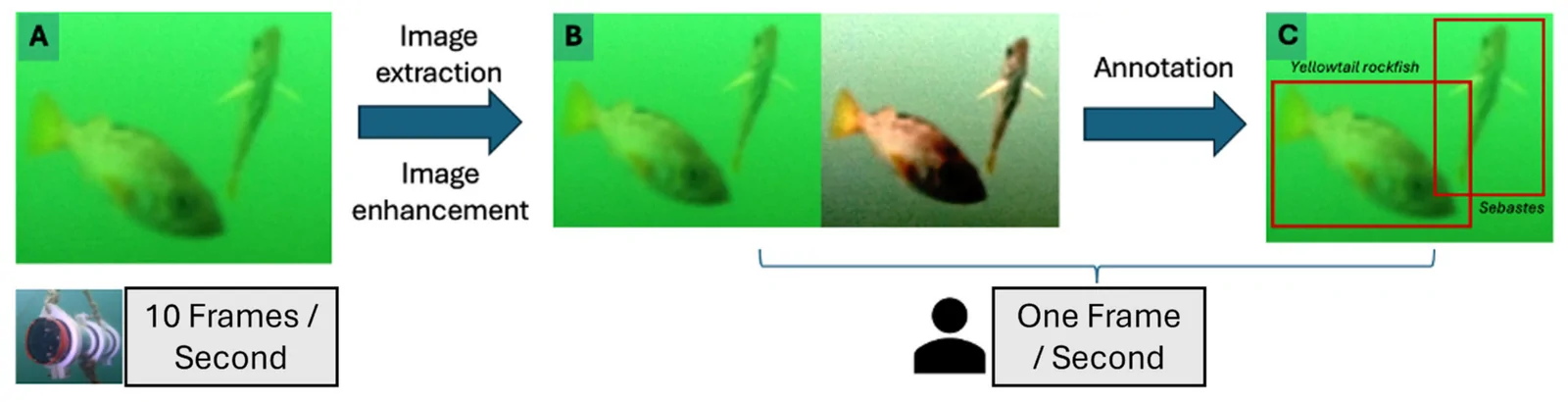
Videos were recorded by FishCams at a rate of 10 frames per second (**A**). We extracted images at a rate of one frame per second, and used an image enhancement algorithm to remove turbidity and discolouration effects from the image (**B**). Annotators then reviewed and annotated animals of interest within the image (**C**).

**Figure 6:**
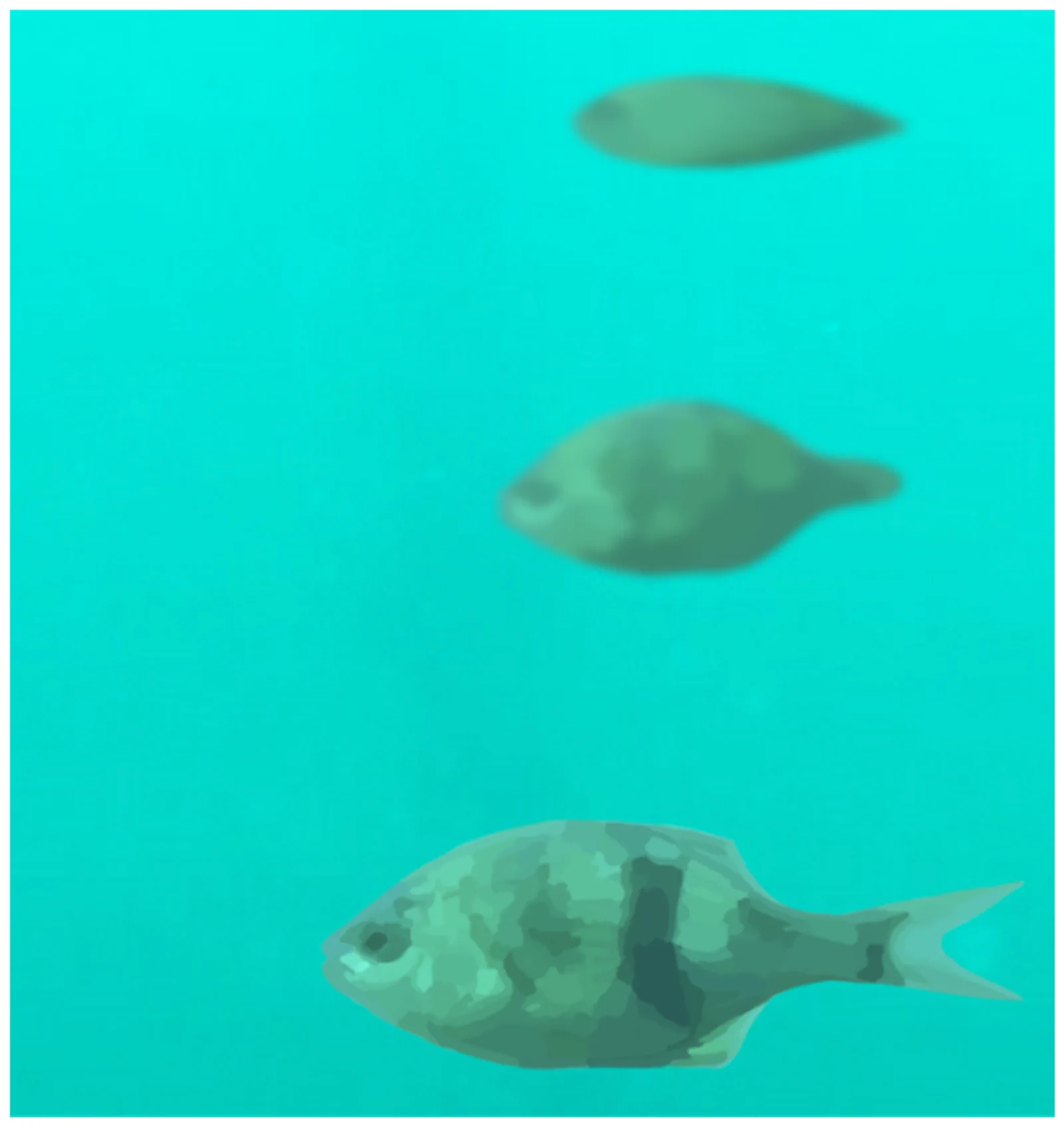
Due to identification challenges posted by turbidity, we annotated organisms to various pseudo-taxonomic levels that were determined based on visually distinguishable features. This illustration demonstrates how one species of fish (pile perch, *Phanerodon vacca*) may be identified to three different taxonomic levels depending on the clarity of the image: class-level (Actinopterygii, top), family-level (Embiotocidae, middle), or species-level (*P. vacca*, bottom). The taxonomic levels (e.g., species, genus, family-level) to which we identified each animal varied depending on visually distinguishable morphological features and were revised over the course of the project based on expert consultations and review of the relevant literature. Artist credit: Tom Zhang.

**Figure 7:**
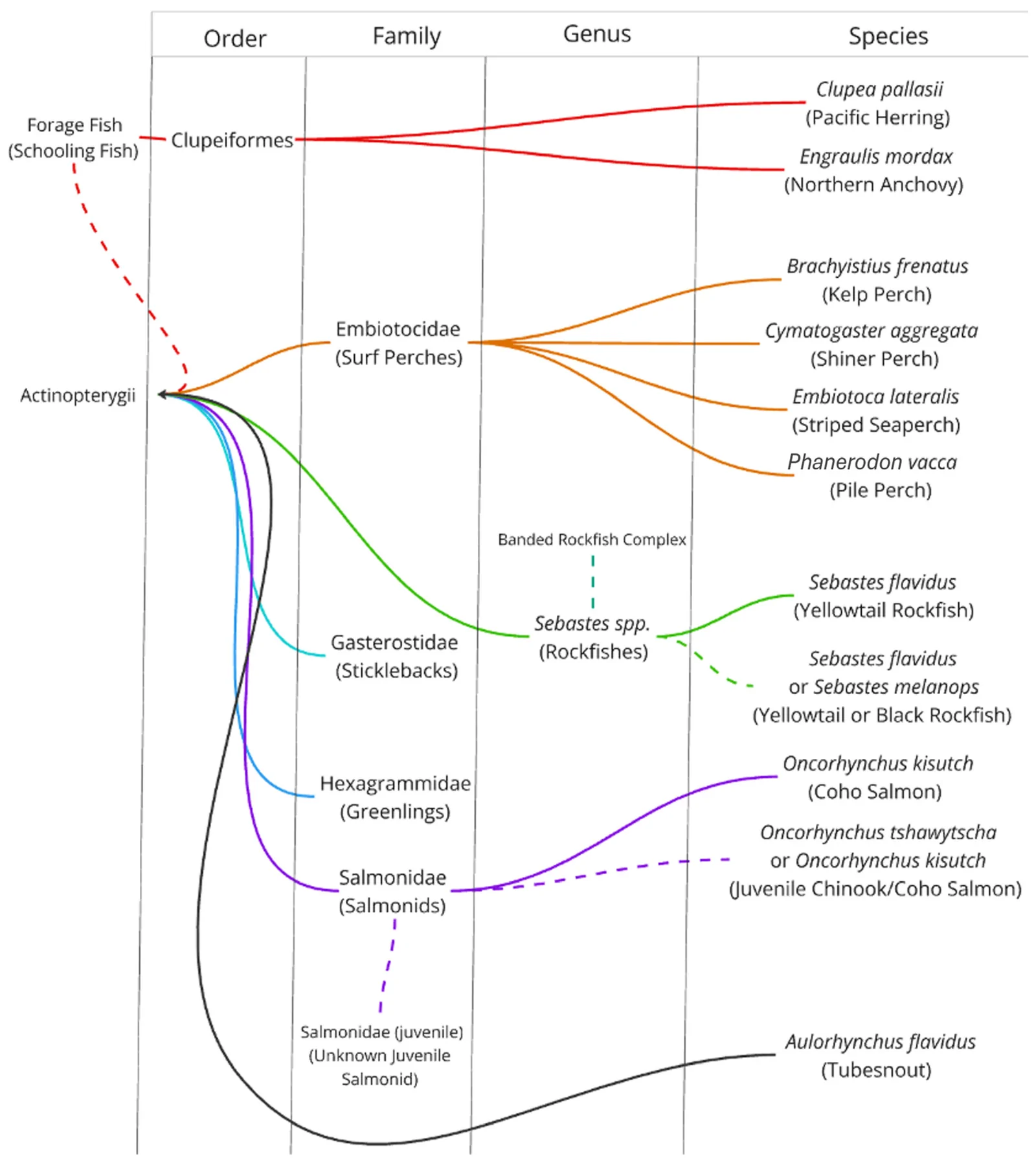
Hierarchical tree containing each label for fish groups seen in the training image dataset. Solid lines indicate linkages between taxonomic groups, and dotted lines indicate visually similar (pseudo-taxonomic) groups.

Annotations were made in a built-for-purpose dashboard using Python (version 3.X) software (predominantly the Open-CV library; [22]). Each annotation consisted of a rectangular bounding box, drawn as closely around the organism as possible, and assigned an identification label (e.g., Actinopterygii). Images were initially annotated at a high frame rate (every 10 frames (~1/3 of a second) before the use of screening algorithms described below) and used an image-enhancement algorithm [23] to reduce the visual effects of turbidity (Figure 5). To maximize consistency in our annotations, we established a series of rules:

(a) Organisms were identified solely by the information within each image (i.e., no context from previous or subsequent frames were used). For example, a fish may initially be identifiable only at the superfamily level when distant; as it approaches the camera, additional features may allow for identification at the family, genus, or species level (Figure 6). For the purposes of validating the 1 fps frame sampling rate, when a fast-moving organism appeared in footage but was unidentifiable to a high taxonomic level, annotators watched the full-length (10 fps) video in which the organism appeared. No new taxa were identified using this process during the study period.
(b) Each organism was identified to the highest taxonomic resolution possible. For example, if an organism displayed characteristics of two different species in a genus, the annotation was made at the genus level. We established visually similar groups for animals that consistently appeared morphologically similar and could not be easily differentiated (e.g., lion’s mane jellyfish (*Cyanea capillata*) and fried egg jellyfish (*Phacellophora camtschatica*)). For the purposes of discussing results in this study, groups that were established based on visual similarity and those established based on taxonomy are henceforth referred to as ‘pseudo-taxa’. A hierarchical representation of fish pseudo-taxa in the classification system is shown in Figure 7, with the full hierarchy shown in Figure S1 and summary statistics provided in Table 1 (fishes) and Table 2 (gelatinous zooplankton).
(c) Partway through the project, it became evident that some groups (e.g., unknown forage fish, shiner perch (*Cymotogaster aggregata*)) appeared in footage far more frequently than other pseudo-taxa. To maximize annotation efficiency, once a group had approximately 2000 annotations, we no longer drew bounding boxes around the organism if it was the only object of interest in the image. This boundary was approximate and selected because we estimated that an animal should not require more than 2000 annotations to achieve high model performance (based on estimates from Ditria et al. 2020 [13]). Annotations for some of these groups still exceeded 2000, however, because we were still required to annotate animals if they appeared concurrently with other groups of interest. This requirement is needed because, to use an image in model training, all animals of interest in the image must be annotated.
(d) When annotating schooling fish, we assumed that all fish within a given school were of the same species. Consequently, all fish in a school were identified to the same taxonomic level as the highest-taxon annotation assigned within that school in each image. For example, if a school of fish included one individual identified as Pacific herring (*Clupea pallasii*), all fish in that school were annotated as *C. pallasii*. Fish were considered part of the same school if they had similar body shapes, were oriented in the same cardinal direction, and there were more than three individuals clustered within approximately one quarter of the image. This was a reasonable assumption based on annotator observation, as we manually reviewed over 100 schools detected during the project, and out of these schools there was only one frame in which multiple forage fish taxa were confirmed (a single Northern Anchovy, *Engraulis mordax*, in a school of Pacific Herring, *Clupea pallasii*).

##### Deciding on Scope and Pseudo-Taxa Included in Annotations

Our primary annotation objective was to develop a method for efficiently collecting training data to broadly monitor macrofaunal biodiversity. To achieve this, we manually annotated any clearly identifiable living animal in the footage, assigning each organism the highest possible taxonomic resolution based solely on its morphological features. Although annotating all animals was not initially within the project scope, the early observation that >90% of the footage contained no animals enabled this approach.

Our computer vision model required that annotations be based solely on the morphological information available in each image, which was further complicated by the video colouration and resolution challenges previously described. To overcome these challenges, we adopted a multi-step approach by annotating at multiple taxonomic levels. When a new taxon first appeared, identification was made by consulting the relevant literature (e.g., identification guides) and local experts, with external expert opinion sought when necessary. On three occasions, strategic identification meetings were held with relevant experts for specific pseudo-taxa (rockfish, forage fish, and gelatinous zooplankton) as part of the quality assurance and control process. Once an organism’s identity was confirmed, annotators examined adjacent frames to document distinguishing features of that taxon, and opportunistic video playback was used to confirm potential new species or ambiguous identifications.

To ensure consistency across annotators, we maintained a live document called the ‘annotation label record’ that listed hierarchical identification features for each taxon. This document was essential because common identification features were often not visible in the footage, organisms appeared seasonally, and patterns for uncommon pseudo-taxa emerged only after multiple observations. The document standardized annotation quality and facilitated communication between computer engineers and ecologists. Regular discussion between computer engineers and ecologists in the project was key to success as we often found that technical term definitions, reporting procedures, and analysis standards differed widely between disciplines. Group names were periodically added or revised following routine reviews by ecological experts and the supervising annotator, allowing for a detailed record of significant changes throughout this process.

#### 2.2.4. Step 3: Salient Motion Filter

To address the large volume of image data requiring annotation and the absence of initial annotations to train a supervised detector, we employed a salient motion filtering algorithm (Figure 4). This filter measured the correlation between moving regions in an image (determined by frame differences) and saliency (quantified through statistical information such as brightness or RGB values within image regions). The algorithm served two primary purposes: (1) to select images containing moving objects distinct from the background and (2) to present only frames with salient motion (i.e., a high correlation between motion and saliency) to annotators, thereby increasing the number of positive annotations per unit effort.

Initially, the filter was overly sensitive and frequently detected small background movements, periods of high turbidity, and motion from the farm, which were often in the camera’s field of view. As a result, a high proportion of ‘background’ images (i.e., frames without animals) were shown to annotators. To mitigate this issue, we implemented the salient motion filter as an iterative process, beginning with a highly sensitive setting (maximizing recall and displaying many irrelevant images), then gradually reducing sensitivity based on annotator feedback to optimize the balance between recall and efficiency.

#### 2.2.5. Step 4: Integrating an Image Classification Model (ResNet-18)

Once sufficient annotations (i.e., several hundred images, determined by comparing accuracy scores on a validation dataset) were obtained through the salient motion filter, we trained a binary image classifier (ResNet-18) to more accurately screen out images containing only the background (step 4 in Figure 4). The classifier tried to predict which frames contained an object of interest (i.e., an animal or algae) and which contained only non-target entities (i.e., kelp, farm infrastructure, or marine debris). The annotator would then only view the frame with a prediction confidence greater than 60%. The prediction confidence value was selected iteratively based on annotation feedback; we started with a lower prediction confidence to ensure the classifier was not missing new animals of interest, and raised it iteratively based on the number of false-positive (i.e., ‘empty’) images the classifier presented.

This step greatly improved the efficiency and accuracy of our object detection process, allowing for faster annotation of the large dataset by focusing annotator efforts on relevant frames. New annotations created using the outputs of this filter were periodically added to the training dataset for the ResNet-18 classifier. This approach allowed for the model to be up to date with the seasonal changes to both the seaweed farm (i.e., infrastructure and seaweed growth) and appearance rates of organisms. When a new pseudo-taxon appeared in the dataset, a new group was created in the annotation label tree (Figure 7) and a bounding box assigned to that label was drawn around the organism.

#### 2.2.6. Step 5: Boosting Annotations Using an ‘Adjacency Filter’

After completing steps 1–4 above, we realized that many pseudo-taxa of interest, particularly exemplar Pacific herring, would not have sufficient annotations to train our supervised detector by the end of our project’s timeline. Up to this point, only every 10th frame from the video dataset had been shown to human annotators. As a result, we created an algorithmic filter that selected frames immediately adjacent to those previously annotated with pseudo-taxa of interest (i.e., animals that did not yet have ≥200 annotations), which we used to supplement annotations for uncommon or rare pseudo-taxa. This was primarily done for the Pacific Herring taxon, as this group was deemed important to include in model output results. The annotations made using this filter could then be added to the rest of the training dataset for the supervised detector.

#### 2.2.7. Step 6: Training a Preliminary Object Detection Model (YOLOv5)

Using the supplementary annotations from the adjacency filter, we trained a preliminary YOLO model (YOLOv5). YOLOv5 was used during this step as YOLOv8 had not yet been released during the conceptual phase of the project, and YOLOv5 had previously been trialed on large ecological datasets (e.g., [18,24]). Like the ResNet-18 classifier above, the YOLOv5 model was a supervised model trained on existing annotations, used to further refine the process of identifying images with positive annotations and showing only those images to annotators. While the ResNet-18 classifier was used only to ‘classify’ images of interest (hence the term ‘binary classifier’), the YOLOv5 model marked where and what previously labeled pseudo-taxa were within each image (though bounding boxes for detections were not made visible to annotators). This object detection model provided a more efficient and accurate method for detecting pseudo-taxa in the video dataset and was the final refinement step in video footage that was shown to annotators.

#### 2.2.8. Step 7: Quality Assurance and Quality Control (QAQC)

After annotating all selected frames (March 2022–November 2022), quality assurance of the resulting training dataset was conducted using custom-built image review software modeled after the ‘Largo’ tool in BIIGLE [17,25]. The supervising annotator systematically reviewed all bounding boxes drawn by the annotation team, cross-referencing each image with the established morphological features recorded in the Annotation Label Record. Erroneous or ambiguous bounding boxes were reassigned or discarded to ensure accuracy and consistency; while we did not track the precise number of erroneous annotations, this step was important given that we revised our annotation criteria for some taxa during the project (e.g., based on new information from expert reviewers).

This process resulted in 241,000 annotations across 54 pseudo-taxonomic categories. To ensure that the final model was ultimately trained on taxa with sufficient numbers of annotations, we set a minimum threshold on the number of annotations required for a group to be used in the final model training (minimum 200 annotations per group). Pseudo-taxa that were identified by annotators but did not meet this threshold were aggregated to their respective parent groups. As a result, 23 pseudo-taxonomic groups were used in training the final YOLOv8 object detection model.

#### 2.2.9. Step 8: Training the Final Object Detection Model (YOLOv8)

Before training YOLOv8 on our annotated training dataset, we reserved 10% of all images, with representatives from all categories, to test our output model. We did not reserve any images for validation as we opted not to edit or fine-tune hyperparameters or model selection for our dataset. We opted not to tune hyperparameters because of the large dataset size relative to available compute resources, and therefore iterative tuning was not practical to align with the project timeline.

Despite the use of the refinement algorithms described above, most images in our annotated training dataset were ‘empty’, meaning the image contained open water (or water with seaweed farm infrastructure) with no organism detections. Including all of these ‘negative’ annotations would bias our model to result in false negatives. To combat this, we limited our final training dataset to include only 33% randomly sampled negative annotations from the total number annotated (i.e., only one third of ‘empty’ images were used to train the final model). This sampling threshold was found by iteratively reviewing training dataset scores, and finding a composition of positive and negative images that resulted in an ideal balance between precision and recall.

Once all annotated image data for the specified period of the study (March to November 2022) were reviewed by the supervising annotator (>240,000 annotations, >400,000 frames), we trained the YOLOv8 model to identify and count all pseudo-taxa of interest that were identified during the training period. We chose YOLOv8 due to its advanced capabilities in handling large datasets and its proven accuracy in real-time object detection tasks (e.g., [26,27]). MaxN was chosen as the abundance metric for predictions, which uses the maximum number of individuals for each taxon or visually similar group found in a single frame within each video, and is a common way of conservatively estimating abundance from underwater footage [28]. MaxN abundances were then aggregated at higher time series (e.g., daily or monthly) as needed for statistical comparison. Once trained, YOLOv8 was applied to identify and count macrofauna of interest on all footage collected by the FishCams for the parent project (March 2022–June 2023).

#### 2.2.10. Step 9: Validation

##### Step 9a: Validating the Model Dataset (i.e., Predictions from YOLOv8)

To validate our model, we evaluated the performance of the trained YOLOv8 model on the test data for its precision, recall, mean average precision, and area under the receiver operator curve (ROC) [29,30]. Precision measures the proportion of detected objects that were correctly identified, reducing false positives (True Positive/(True Positive + False Positive)). Recall quantifies the proportion of actual objects correctly detected, minimizing false negatives (True Positive/(True Positive + False Negative)). Mean average precision (mAP) evaluates the model’s accuracy across different confidence thresholds by averaging precision-recall curves for all classes. The area under the ROC plots the True Positive rate and False Positive rate for a model across all confidence thresholds (i.e., how sure the model must be in its answer before making a prediction). The ROC is used as a summary of the model’s ability to distinguish between positive and negative cases across varying thresholds, with higher values indicating better classification performance. These metrics help to validate the trained YOLOv8 model so it can effectively detect and classify pseudo-taxa within our dataset.

We also used the relative mean F1 score for all classes evaluated over different confidence thresholds to determine an optimal threshold, as this is a common metric for evaluating performance in CV datasets [31,32]. F1 serves as a suitable ‘balance’ between precision and recall, and is defined as follows:

F1 = 2 × ((P × R)/(P + R))

where P = precision and R = recall.

To refine our model predictions, we determined an optimal threshold to maximize the average F1 score of all classes. The results of this evaluation can be found in the form of a confusion matrix, precision-recall curve, and raw outputs of the precision and recall values for each class (with mAP50 and mAP95) in Section 3.1.2.

##### Steps 9b/9c: Validating Community Representation

A limitation of YOLOv8, as with other deep learning models, is that it can only identify and count pseudo-taxa with sufficient annotations in the training dataset, which can vary widely by group [14,17]. As discussed above, we set an arbitrary threshold of 200 annotations for groups to be considered in the model. Consequently, rare species, seasonal or migratory species absent during the annotation period, alternate morphotypes (e.g., breeding or juvenile vs. adult forms), and higher taxonomic groups not included in the dataset were unrepresented in outputs from the model. Therefore, to ensure that the training dataset adequately represented the breadth of fish and gelatinous zooplankton community diversity during the study period, and to explore what rare pseudo-taxa may be missed, several validation steps were adopted.

**Figure 8:**
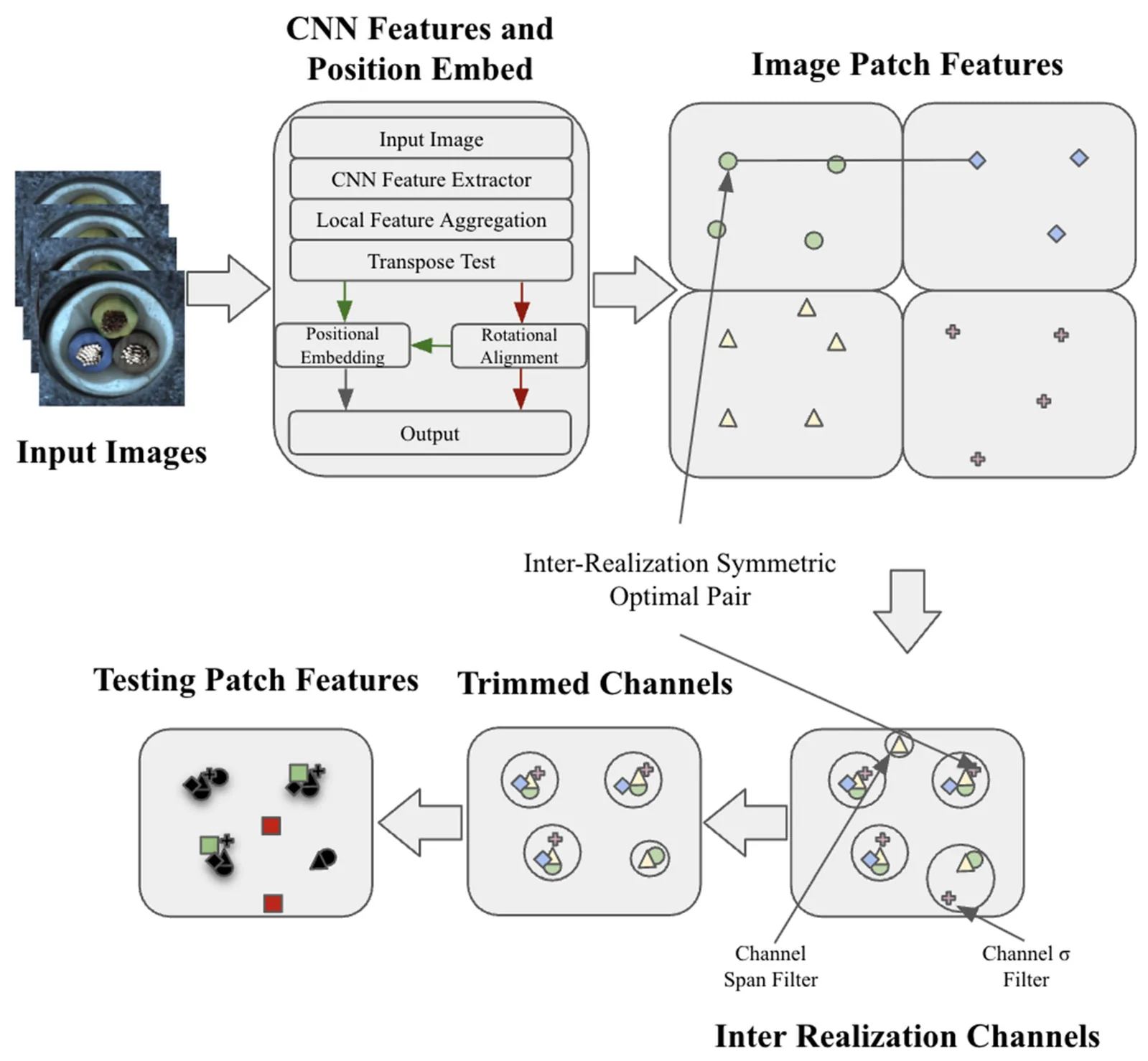
Diagram of information and processing pipeline for Online-InReaCh (credit: Declan McIntosh, adapted from McIntosh and Albu 2023 [33]). Points in the figure indicate patch feature embeddings, circles show channel membership.

First, a novel unsupervised filter algorithm, Online-InReaCh, was employed over the entire study period [33]. Online-InReaCh dynamically models nominal visual features captured in the FishCam videos and detects deviations from this model. The filter selected 200 images of interest per FishCam deployment that contained detected anomalies, which were then reviewed by the supervising annotator. Given that fishes and other organisms are relatively rare in the video stream, they appear as anomalies. However, not all detected anomalies corresponded to fish; some included marine debris or, most commonly, camera encoding glitches. Detected anomalies’ component image patches are encoded in R1024 feature vectors using intermediate feature maps of a pre-trained convolutional neural network. Specifically, Wide-ResNet-50, with weights published by PyTorch (version 2.X), was selected due to its widespread use in anomaly detection [34]. The patch feature vectors of the detected anomalies form a distribution in this feature space. Distances between vectors in this feature space can be used as a measure of visual dissimilarity between two patches of visual features. From this distribution, visually distinct detections are sampled by maximizing the distance between them, ensuring a representative set of rare, visually unique detections. This process is illustrated in Figure 8 and detailed in McIntosh and Albu (2023) [33].

Second, a pseudo-random subset of videos (equivalent to a day’s worth of video per camera, site, and month) was annotated without algorithmic filters to validate the decision to use screening filters and to cease annotation after November 2022. These images were annotated using the BIIGLE program [25] and the same annotation rules as described above, but without the use of the algorithmic screening filters. Lastly, annotators reviewed opportunistic videos from the unannotated period (December 2022–June 2023). All three methods yielded no new pseudo-taxa of interest, which was expected given the project’s cautionary approach and the high sensitivity of the algorithmic filters during the initial phases.

Despite the validation methods described above, a byproduct of the workflow described above is that non-equal effort was used in annotation of footage over the project’s duration (e.g., due to the use of sequential algorithmic filters), which likely influenced the type and number of annotations of pseudo-taxa represented in this dataset. As a result, annotation data should not be used as a true ecological representation of the relative abundance of each taxa.

#### 2.2.11. Step 10: Final Training Dataset and Interpretation of Predictions

**Figure 9:**
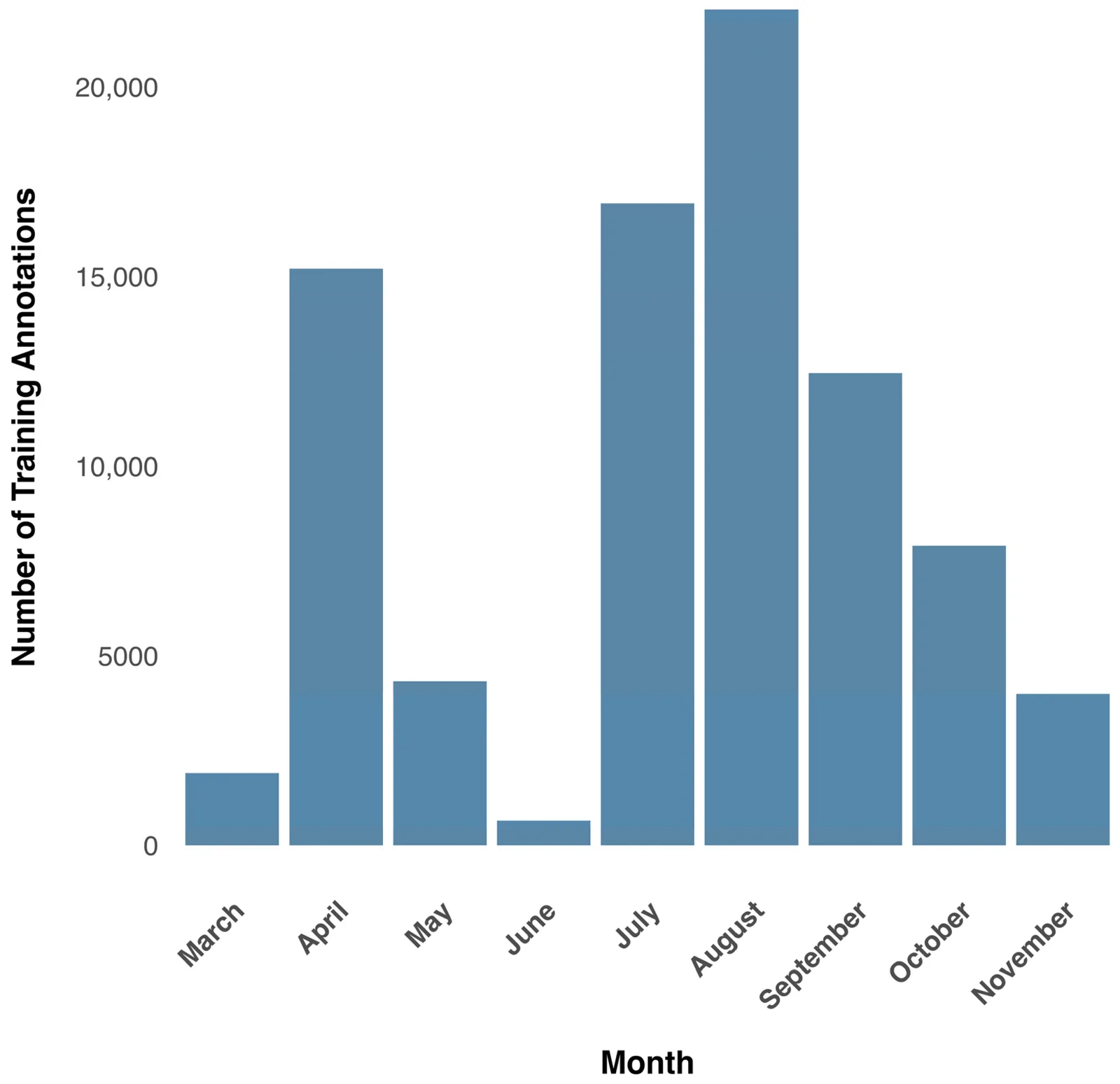
Monthly annotations (excluding background) used to train the YOLOv8 object detection model. A camera malfunction prevented annotations from being recorded for the majority of June 2022.

**Figure 10:**
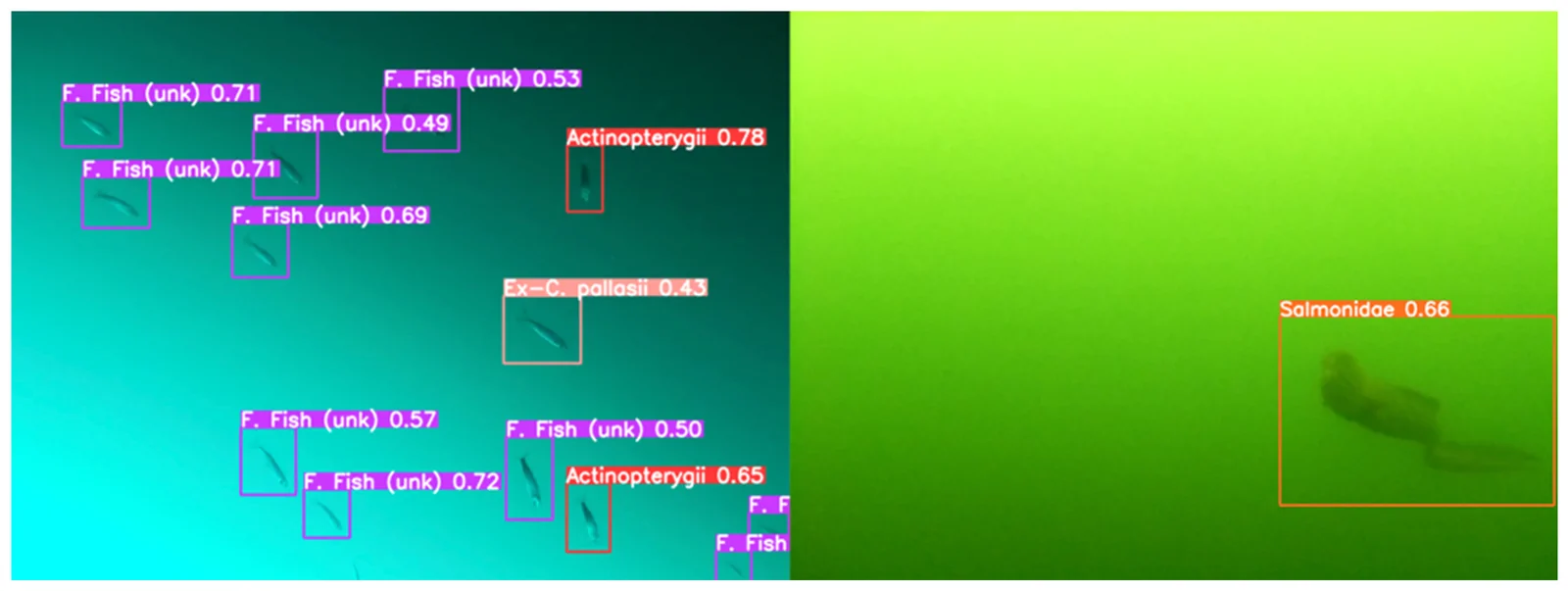
Example frames from FishCam video with YOLOv8 detection boxes showing predicted identity of an entity within the box, and percent confidence in the quality of the detection. The left image shows accurate predictions (schooling fish, “F. Fish (unk)”, and a Pacific herring, “Ex-C. pallasii”), and the right image shows an example of an erroneous prediction on a rare taxon from an earlier model iteration (predicting a wolf eel as “Salmonidae”).

The final YOLOv8 detector was trained on a dataset comprising approximately 241,000 annotated images containing 49,627 fish and 19,835 gelatinous zooplankton annotations across 54 categories. Negative (i.e., ‘background’ or ‘empty water’) images were selected randomly and uniformly across the study period based on the available set of negative ground-truth images. The resulting dataset of model predictions was then reviewed opportunistically by annotators, in addition to the evaluation steps described in Section 3.1, to evaluate performance. Quantitative evaluation of the annotation data should be interpreted with caution, as sampling effort for some highly abundant pseudo-taxa was tapered once sufficient annotations were available to train the supervised CV detector, which, in combination with other factors (camera fouling, equipment failure), resulted in an uneven sampling effort across the study period (Figure 9). The outputs of the supervised detector are more reliable for evaluating population patterns of abundant pseudo-taxa (e.g., Clupeiformes, Figure 10). The results from this process formed the final dataset used for subsequent analysis, as detailed in Bates et al. [19].

### 2.3. Assessing Relative Model Performance by Annotation

After assessing model performance on the collected training dataset, we investigated the relationship between model performance and the number of annotations used in model training. To do this, we grouped our dataset into 10 of the most common parent pseudo-taxa and re-trained the final YOLOv8 model at eight different levels of annotations for each taxon: 25, 50, 100, 150, 200, 250, 500, 1000, and the maximum number of annotations per taxon. The sample size for each taxon included in the experiment is shown in Table 3.

We subset the original dataset into 10 taxonomic groups, re-labeling the annotations for each group appropriately (Table 3). We used this approach because most groups in the original dataset had fewer than 500 annotations and grouping them taxonomically would provide more training data for each group in the experiment while keeping annotations at an order of relevance consistent with our annotation rules. We also wanted to maximize the relevance of this experiment to other ecologists looking to train computer vision models on underwater imagery, and we believed that creating general taxonomic groupings would help that endeavor. The final dataset used in this experiment is shown in Table 3.

In total, 58,000 annotations were used to train the object detection model. Most of the dataset was comprised of forage fish (>24,000 annotations, 41%), Embiotocidae (24%), and hydromedusae (18%) pseudo-taxa, while several pseudo-taxa (Salmonidae, *A. flavidus*, drift algae, Ctenophora, *Sebastes* sp.) each accounted for less than 4% of all annotations in the dataset. These proportions reflect the frequency with which each taxon appeared to annotators.

#### Shared Feature Learning Justification

Due to dataset imbalance, some pseudo-taxa had fewer training annotations than others. To standardize model runs and improve potential comparability of this dataset to future studies, we included these low-annotation pseudo-taxa in later runs (i.e., runs requiring ≥500 annotations, even though these taxa did not have enough annotations available). Including these pseudo-taxa also allowed us to assess whether the model could improve its performance through shared feature learning [35]. Shared feature learning occurs when training a single model on multiple groups or more generally tasks simultaneously, enabling it to learn generalized image or other data features (such as edges, textures, and shapes) that apply across downstream categories or tasks. This effect helps the model recognize patterns in underrepresented pseudo-taxa by leveraging better learned lower-level generalizable features from better-represented groups. For example, a CNN trained on multiple fish pseudo-taxa can detect common traits like fin shape and colour patterns, improving classification accuracy or detection localization even for pseudo-taxa with limited training data [24,36]. Studies on feature sharing in CNNs and other learning architectures have shown that features learned in one task can improve model performance in related detection tasks within the same model, such as identifying other types of objects [35].

**Figure 11:**
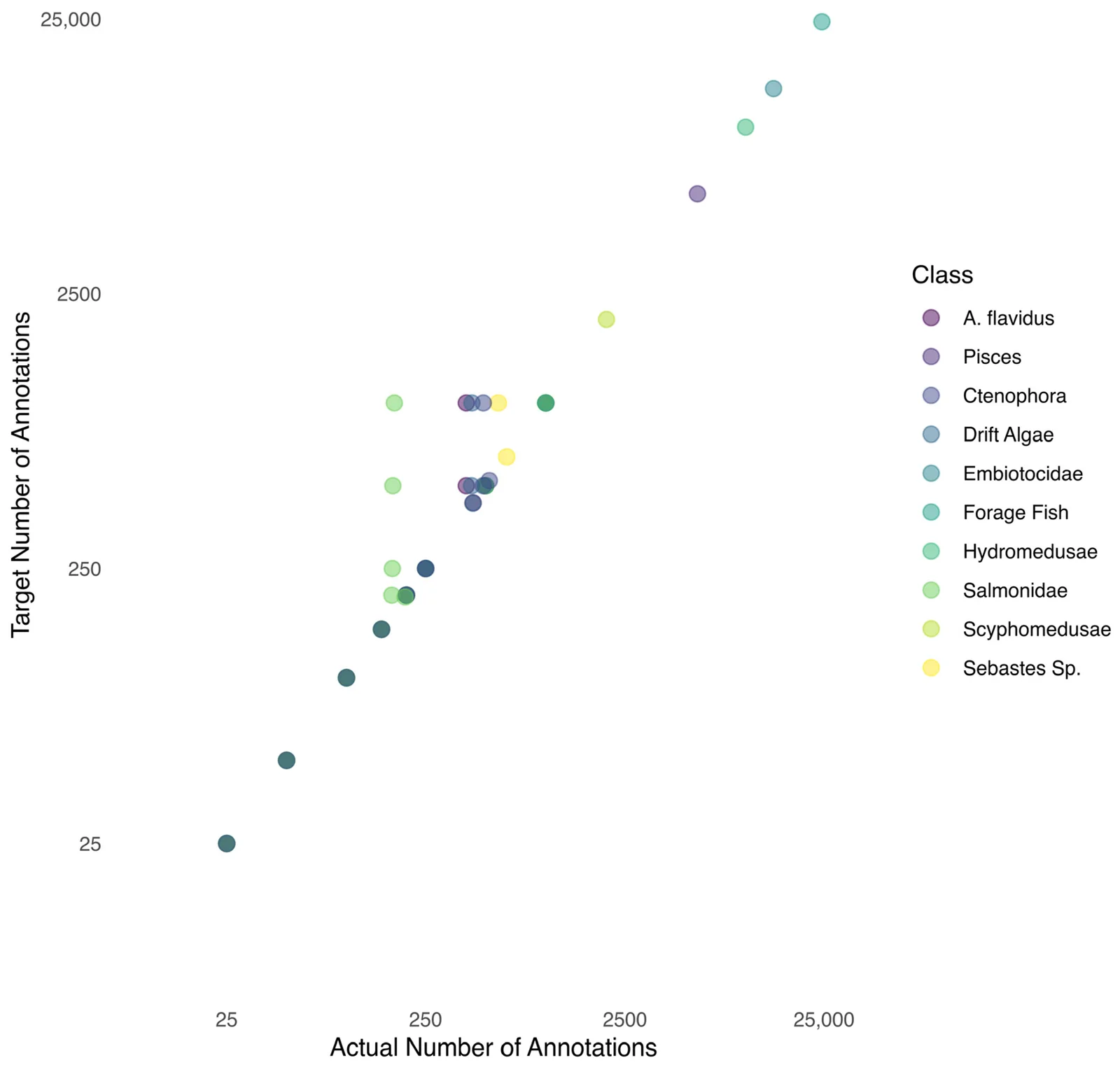
Target versus actual number of annotations for each group considered by the object detection model. Divergence from the 1:1 line indicates that the group has fewer annotations than required for the given model run.

Figure 11 shows the target number of annotations for each model run (*y*-axis) against the actual number of annotations that could be provided for each taxon (*x*-axis). Several pseudo-taxa had insufficient annotations to meet the target for model runs in which ≥500 annotations were required (i.e., the groups had fewer than 500 annotations total). These pseudo-taxa were included in the final model as we were interested in investigating the effects of learning shared features within the dataset, though this unequal distribution in training data should be noted when interpreting results from the experiment. Increased training data typically lead to better model performance, even when this data is imbalanced, which affects the final model run (using all training data). Additionally, several pseudo-taxa were rare (e.g., drift algae, Salmonidae), and as such they did not have sufficient annotations to satisfy some of the model runs (i.e., ≥500 target annotations).

## 3. Results

### 3.1. Experiment 1 Results: Object Detection Across All Pseudo-Taxa

This section contains results from the YOLOv8 model that was trained on the full annotated dataset. The final model was trained on 241,000 images across 9 fish pseudo-taxa and 10 gelatinous zooplankton pseudo-taxa.

#### 3.1.1. Confusion Matrix

##### Fishes

The highest-performing fish groups included *C. aggregata* dark morph (shiner perch breeding males, Accuracy: 0.916, True Positives (TP) = 76), *P. vacca* (pile perch, Accuracy: 0.905, TP = 134), Embiotocidae (surfperches, Accuracy: 0.899, TP = 558), *A. flavidus* (tubesnout, Accuracy: 0.898, TP = 44), Salmonidae (salmonids, Accuracy: 0.897, TP = 26), and *C. aggregata* (shiner perch, Accuracy: 0.891, TP = 459). In addition to having similar accuracy, the model had similar misclassification (i.e., erroneous) pseudo-taxa for these groups. Specifically, almost all misclassifications (i.e., false positives) of the model for fish pseudo-taxa were attributed to the general ‘Actinopterygii’ and ‘Embiotocidae’ groups; when the model mislabeled fish groups, it tended to make errors in assuming the groups were general fish taxa, and did not mistake any fish for gelatinous zooplankton or drift algae groups.

The worst-performing fish groups were Actinopterygii (unidentified bony fishes, Accuracy: 0.610, TP = 379) and unidentified forage fish (Accuracy: 0.719, TP = 2458). Actinopterygii in particular had the highest misclassification rates by the model among fish groups, with notable errors where 9.7% of detections were misclassified as Embiotocidae and 16.7% were assigned to *C. aggregata*. Furthermore, 13.5% of Actinopterygii detections were mistakenly classified as background (meaning that the model thought they were ‘empty’ images).

##### Gelatinous Zooplankton

The highest-performing gelatinous zooplankton groups in the model included *C. capillata* or *P. camtschatica* (lion’s mane or fried egg jellyfishes, Accuracy: 0.970, TP = 32), Ctenophora (Ctenophore jellies, Accuracy: 0.966, TP = 28), *Aequorea* sp. (water jellyfish, Accuracy: 0.945, TP = 835), and *A. labiata* (Moon Jellyfish, Accuracy: 0.884, TP = 61). *C. capillata or P. camtschatica* demonstrated the highest classification accuracy, with only 3% misidentified as the Scyphomedusae group. *Aequorea* sp. was also among the most accurately classified, with notable misclassification as Cnidaria (11.6%). *A. labiata* was also frequently misclassified as Cnidaria (10%) but otherwise had a relatively high accuracy (0.88).

The worst-performing gelatinous pseudo-taxa, ranked from worst to best accuracy, included Cnidaria (unknown cnidarian jellyfishes, Accuracy: 0.60, TP = 36), *P. bachei* (Lobate Ctenophores, Accuracy: 0.692, TP = 27), and unknown gelatinous zooplankton (Accuracy: 0.695, TP = 339). As with fish pseudo-taxa, the lowest-performing groups tended to be confused with the most general pseudo-taxa and the background; the model frequently misclassified Cnidaria as *A. labiata* (10%), and unidentified gelatinous zooplankton was frequently misclassified as background (21%). As with model predictions for fishes, caution is warranted when interpreting these results due to the limited number of test images, particularly for pseudo-taxa such as *P. bachei* (32 test annotations) and Cnidaria (62 test annotations).

Unfortunately, an unidentified sub-class of gelatinous zooplankton (listed as ‘unidentified gelatinous zooplankton’) was erroneously included in training the model. The results from this group (Accuracy: 0.68) can be wrapped into the parent group ‘gelatinous zooplankton’ for aggregation. The main consequence of including this group in the model is that the overall accuracy for high-level gelatinous zooplankton groups (e.g., gelatinous zooplankton, Hydromedusae, Scyphomedusae) is likely under-reported, as the model needed to account for annotations in this group that were highly similar to those groups.

#### 3.1.2. Precision and Recall

**Figure 12:**
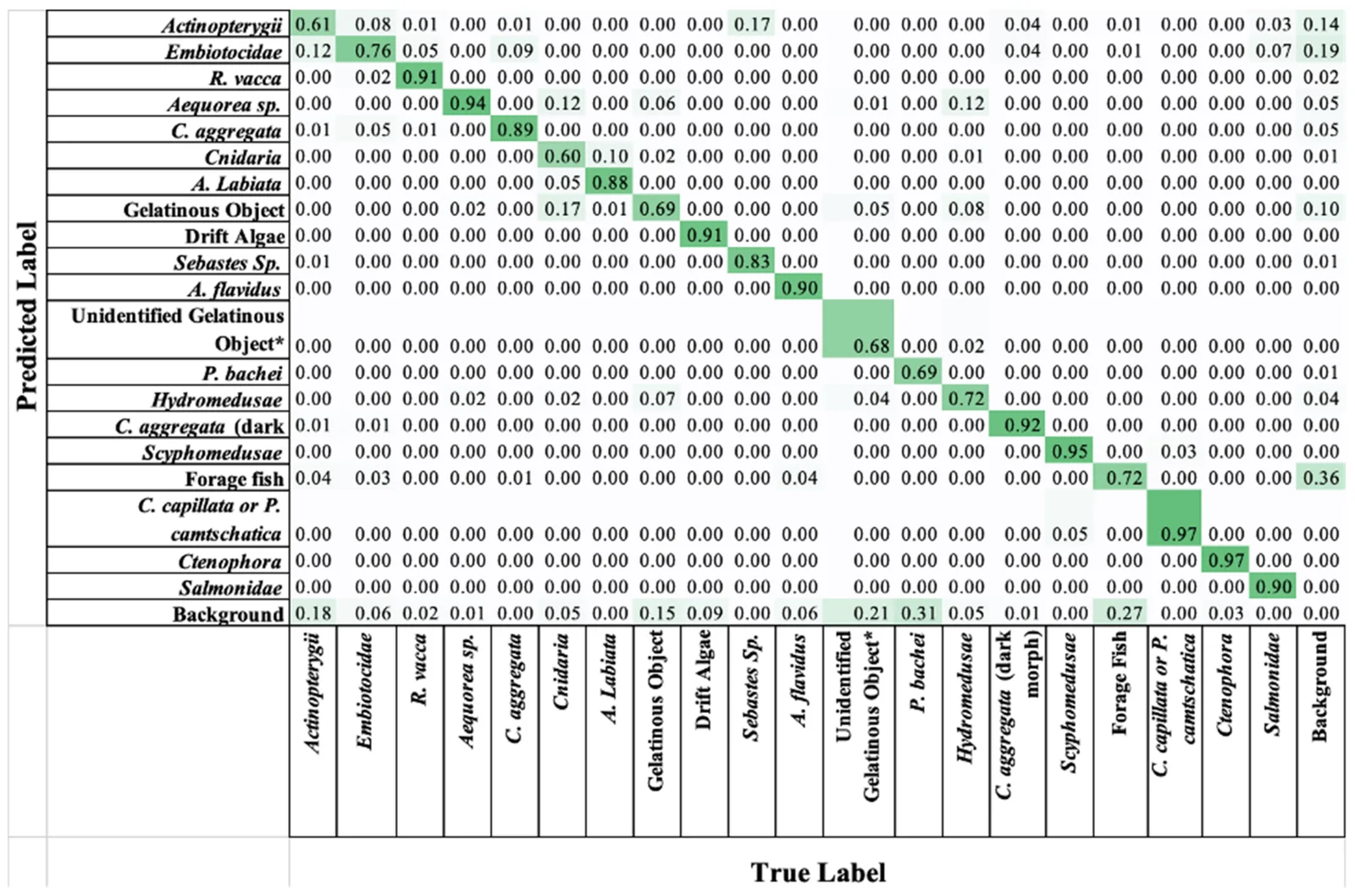
Confusion matrix of the YOLO8 object detection results for each group included in the model. Columns represent the actual annotation label, and rows represent the label that the model predicted for each detection; each column is also normalized, meaning that accuracy and error rates are calculated individually within each column. The group “Unidentified Gelatinous Object*” was accidentally was erronously included in model training; its results can be wrapped into the “Gelatinous Object” group. Green shading is based on relative proportion in each cell (i.e., shading darkens as values approach 1).

Overall, the trained detector performed within expected accuracy ranges, with precision, recall, and mean average precision (mAP) reported at both 0.5 and 0.95 Intersection over Union (IoU) thresholds (Table 4). These results also include outputs from a specialized model trained to distinguish between Clupeidae and *C. pallasii* within the forage fish category. The lowest-performing classifications were broader pseudo-taxonomic groups, particularly Actinopterygii and unidentified forage fish, which were frequently misclassified as background or more specific pseudo-taxa, as shown in the confusion matrix (Figure 12).

**Figure 13:**
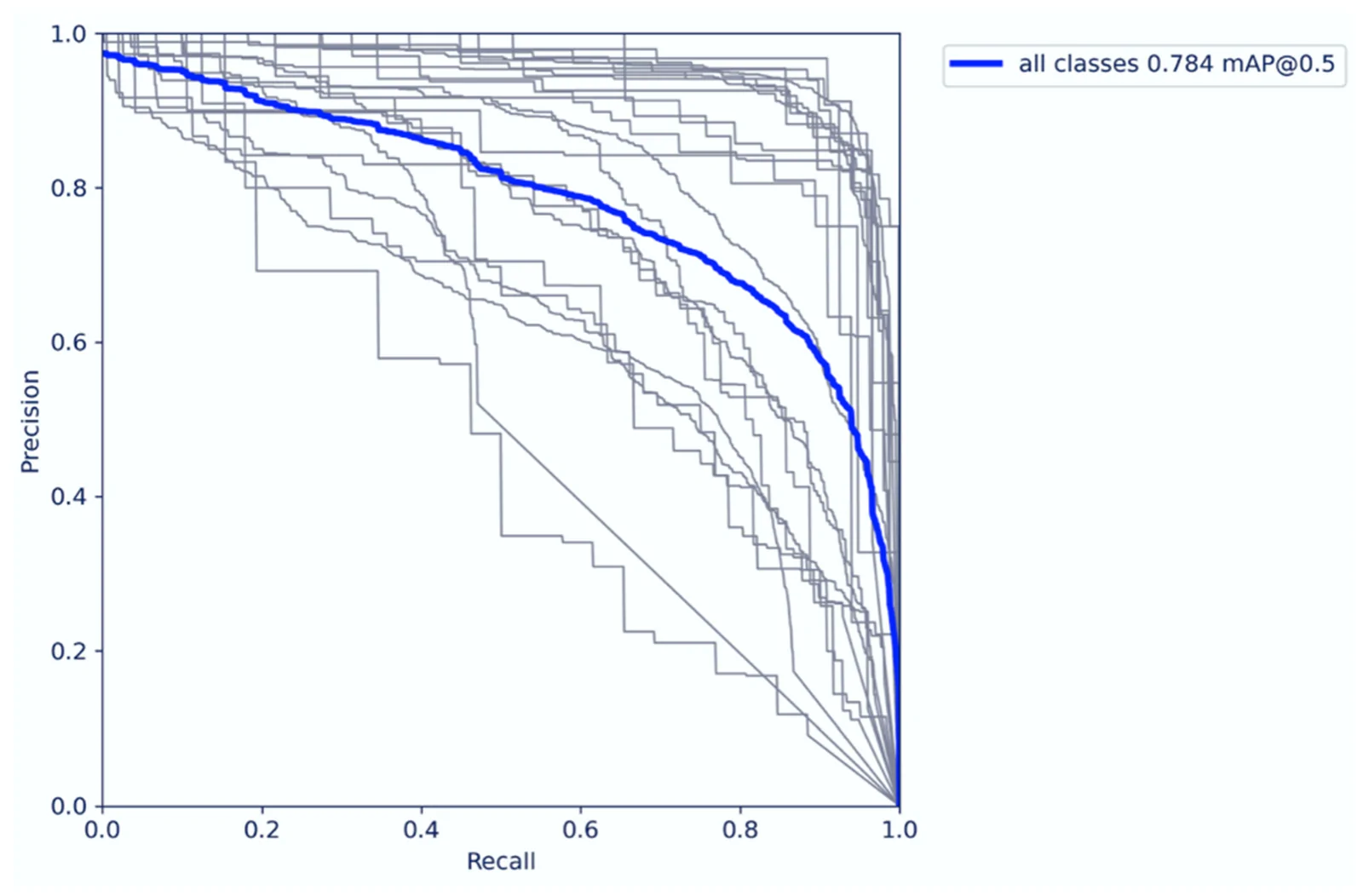
Precision recall curves for all pseudo-taxa for which YOLOv8 was trained using data annotated from FishCams. Grey lines represent individual pseudo-taxa. The blue line is the average precision recall curve, indicating a mean average precision (i.e., accuracy of identification) of 78.4%.

Model evaluation metrics, including precision, recall, and mAP values, were calculated using an optimal confidence threshold of 0.25 (Table 4). Adjusting the confidence threshold from 0 to 1 corresponds to a shift from the rightmost to the leftmost point on the precision-recall curve (Figure 13). The threshold was selected based on the F1 score, which is a standard metric that balances precision and recall to minimize classification errors. Consistent with previous findings, the least precise results were observed for higher taxonomic groups, such as Actinopterygii and unidentified forage fish, which exhibited higher error rates than more specific classifications.

### 3.2. Experiment 2 Results: Re-Training YOLOv8 at Prescribed Annotation Intervals for Each Taxon

**Figure 14:**
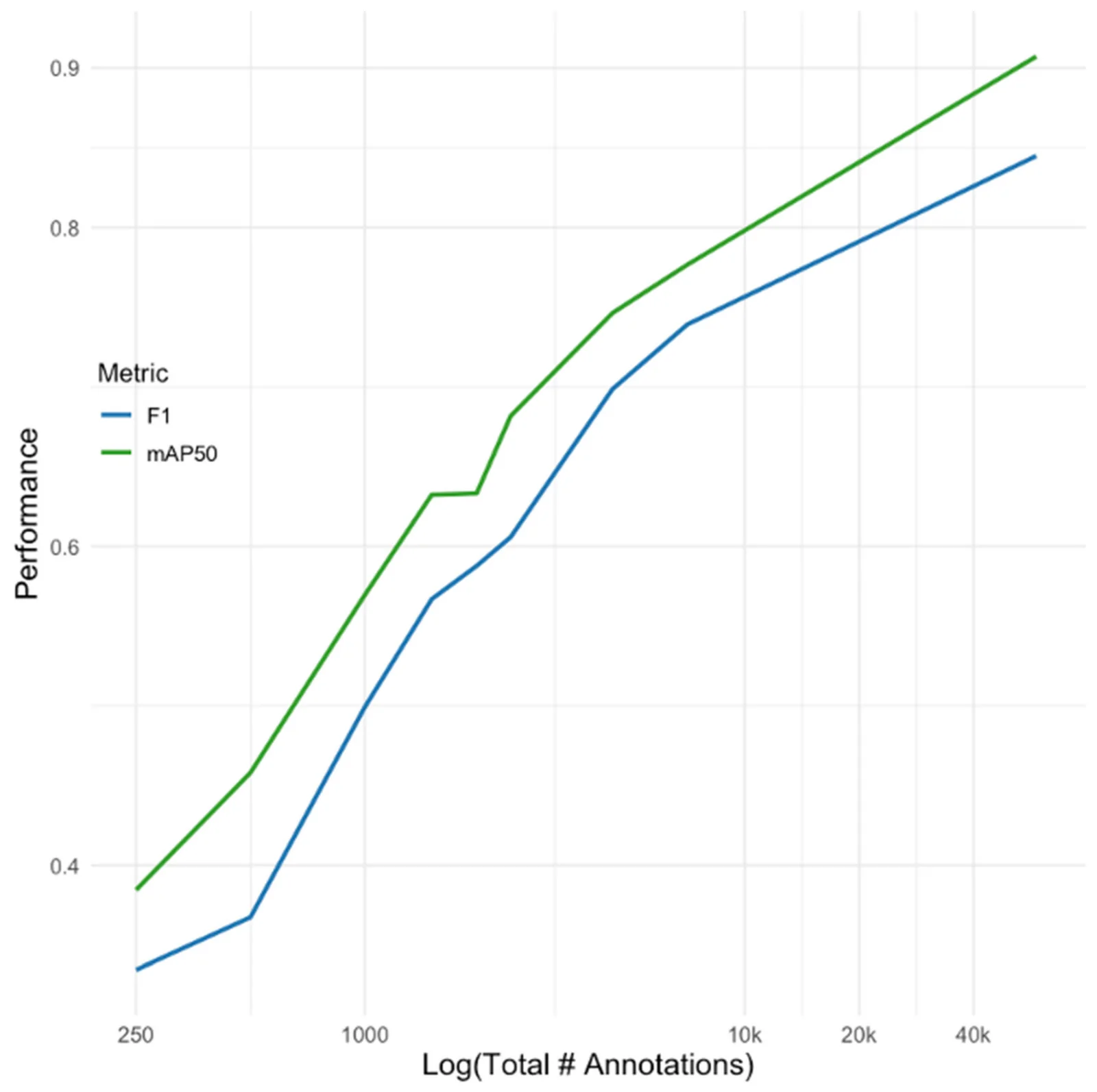
Mean performance (F1) and mean average precision (mAP50) for all considered pseudo-taxa.

Overall F1 model performance for the lowest number of total annotations (250) was 0.33, which improved to 0.84 when the maximum number of annotations (58,495) was used in model training (Figure 14). Mean average precision across all pseudo-taxa followed a similar trend, beginning with a score of 0.38 for the first model run and finishing at 0.91 with the maximum number of annotations. While model performance varied across model runs, F1 and mAP50 did not reach a true asymptote by the final trial, though change in performance reduced as annotations increased.

**Figure 15:**
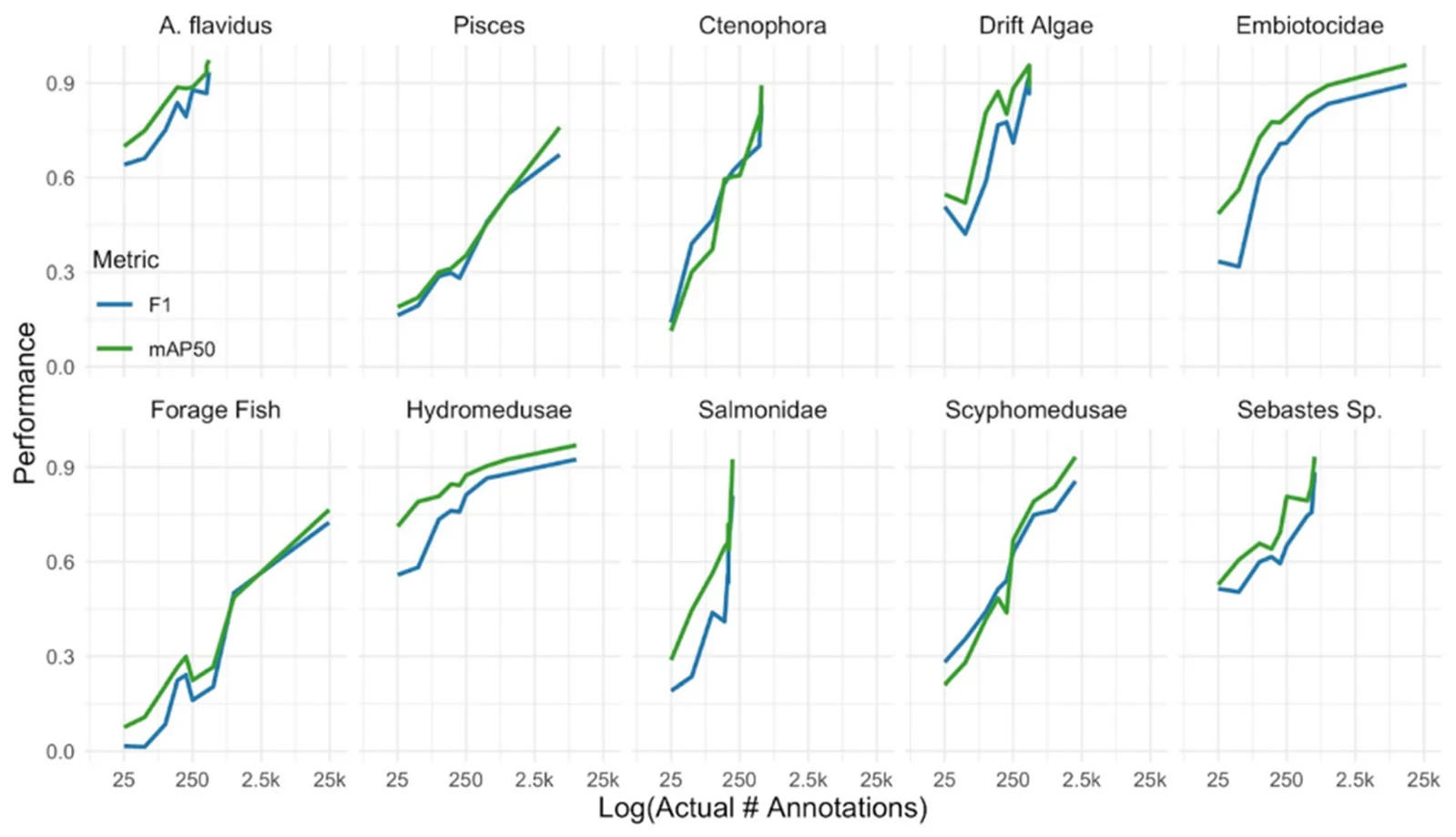
Model performance (F1) and ability to fit a bounding box around an object (mAP50) for each pseudo-taxon, based on the number of annotations considered.

Model performance differed by pseudo-taxa and the number of annotations considered (Figure 15). Some pseudo-taxa (forage fish, Salmonidae, Scyphomedusae) began with a very low performance (F1 < 0.25) and only exceeded 0.9 in the final iteration of the model when all potential annotations were considered. However, other pseudo-taxa (*A. flavidus*, Hydromedusae, *Sebastes* sp.), achieved an F1 performance of >0.50 in the lowest iteration of the model (when considered annotations were limited to 25). Only two pseudo-taxa (Embiotocidae and Hydromedusae) showed evidence of an asymptotic relationship by the end of the model period.

**Figure 16:**
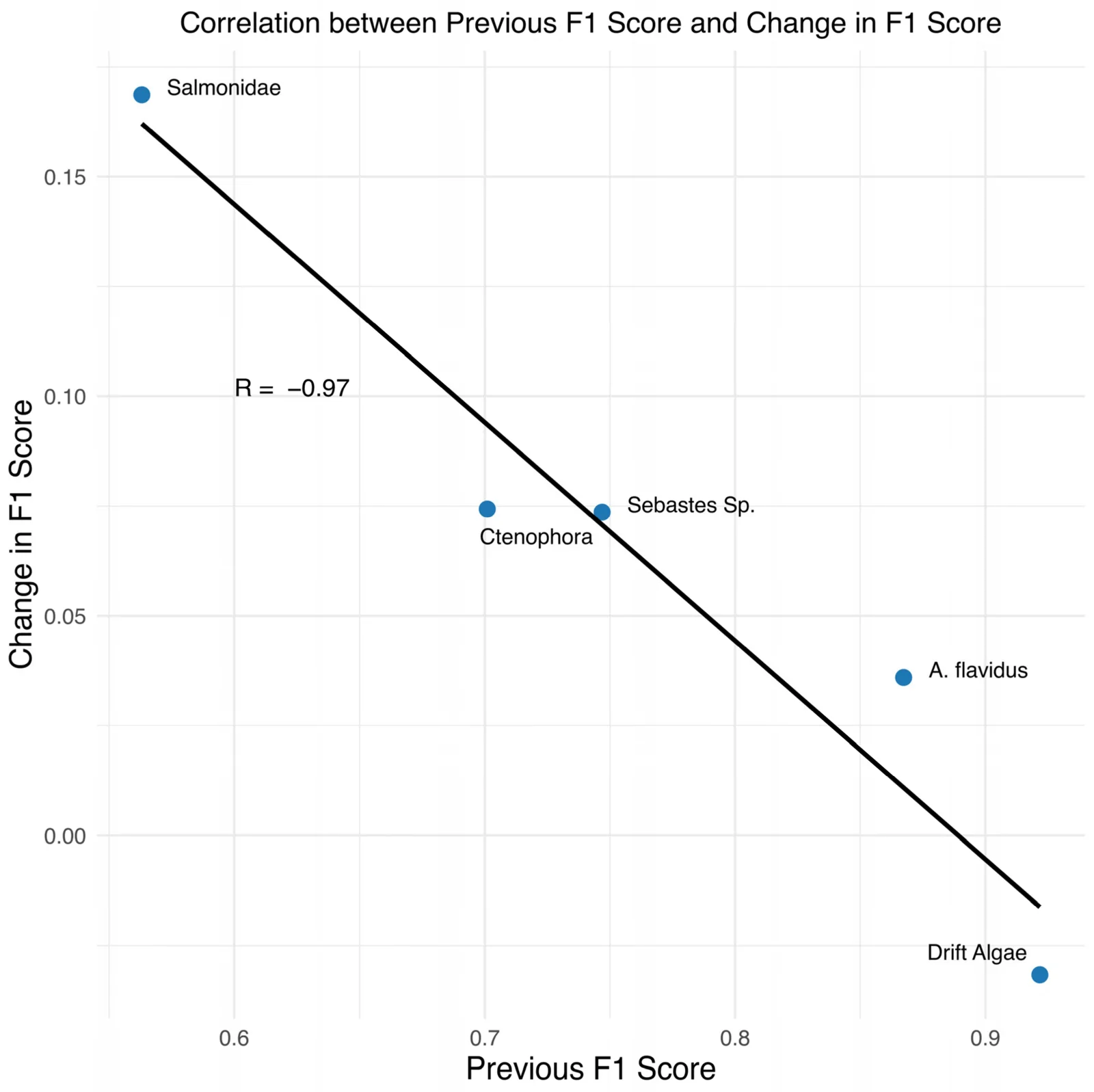
Change in model performance (F1) score as a function of previous F1 scores for groups with the fewest numbers of available annotations. Change in model performance (*y*-axis) indicates the net change in F1 percent for model runs that exceeded the available number of annotations for that group (i.e., any change in model performance is solely reliant on shared learning from annotations of other pseudo-taxa).

Interestingly, the pseudo-taxa with the fewest annotations (i.e., *Sebastes* sp., Ctenophora, drift algae, *A. flavidus*, Salmonidae) continued to show improvement in model runs ≥500 (i.e., runs in which there was little or no increase in annotations for that group). Despite no increases in annotations for the model runs ≥ 500, Salmonidae, *Sebastes* sp., and Ctenophora had the highest gains in F1 performance (0.17, 0.10, and 0.09, respectively; Figure 16).

Model performance improved for several pseudo-taxa in later runs, despite having no additional training images (Figure 16). When examining the change in F1 and mAP50 scores for groups that had already reached the total number of annotations possible, we found a strong negative correlation (R = −0.97) between previous performance and relative improvement in subsequent model runs (Figure 16). This correlation was expected because well-performing pseudo-taxa (e.g., *A. flavidus* and drift algae) would have less room to improve in future model runs. However, we also saw a decline in performance from the highest-performing pseudo-taxa, drift algae (−0.3%).

## 4. Discussion

### 4.1. Experiment 1: Interpreting Results of Training YOLOv8

We examined the performance of a YOLOv8 object detection model, trained based on a novel workflow to annotate images collected from underwater RUV cameras, to identify fishes and gelatinous zooplankton. Overall, the model performed well on lower-level pseudo-taxa (i.e., species- and genus-level) but struggled with more generalized groups (i.e., family-level or higher). Misclassification patterns were often systematic, involving pseudo-taxa with similar shape, size, or coloration, and affected general groups such as unknown forage fish, Actinopterygii, and Cnidaria most of any taxa. Our retraining experiment found that annotation effort was strongly correlated with model performance, though this effect varied widely by pseudo-taxa. We also found that the classification of rare pseudo-taxa (i.e., those with relatively few annotations) can benefit greatly from feature transfer during transfer learning from the ImageNet task, though this effect appears to be negatively correlated with model performance.

#### 4.1.1. Fish Pseudo-Taxa

The object detection model demonstrated relatively high performance (F1 > 0.80) for most fish pseudo-taxa, particularly for groups at the species or genus level. However, there were considerable misclassifications among pseudo-taxa that shared morphological traits, suggesting that errors were systematic and related to visual similarity. For example, pseudo-taxa such as Embiotocidae were primarily misclassified with pseudo-taxa either directly above (e.g., Actinopterygii) or below (e.g., pile perch) the group in our hierarchical tree. This pattern is similar to previous research showing that deep learning-based fish classification models can struggle to distinguish between species with visually similar morphological characteristics, particularly when trained on relatively few annotations [36]. Annotators anecdotally reported that broader groups were more challenging to identify, as they inherently lacked distinguishing morphological features. Consequently, higher-level pseudo-taxa often appeared in blurrier or low-resolution frames and were misclassified as background, which could lead to underestimates of community abundance if not explicitly labeled. This is consistent with ecological studies showing that insufficient taxonomic resolution can result in inaccurate taxonomic assessments [18,37].

We recommend that researchers aiming to estimate fish abundance using CV include at least one broad fish group, in addition to more specific species and genus taxa. Our results indicate that including high-level taxa groupings for fishes is necessary to avoid underestimating abundance, as the majority of fish counted during the project were from pseudo-taxa at the Family level or higher (Table 1). Similarly, our results suggest that studies relying solely on species-level identification in underwater video analyses may underestimate overall fish abundance, as individuals that cannot be confidently identified to species are often excluded from counts. This approach would allow for the higher performance that comes with training a model on species or genus-level pseudo-taxa, while still keeping the more accurate abundance estimate from including a broad-level fish (e.g., Actinopterygii) group. However, caution is warranted when interpreting these results due to the limited number of test images for certain pseudo-taxa, and due to the unequal annotation sampling effort during the study. For example, Salmonidae had relatively few test images (n = 26), which limits potential conclusions about model performance. Future studies with more representative training data may reduce errors for visually variable and uncommon groups while allowing for ecologically relevant abundance estimates, and studies that seek to assess only diversity (rather than abundance of organisms) should focus only on lower-level (i.e., species and genus-level) taxa.

#### 4.1.2. Gelatinous Zooplankton Pseudo-Taxa

*P. bachei*, Cnidaria, and unknown gelatinous zooplankton groups showed higher model misclassification rates than other gelatinous zooplankton groups (Figure 12). Cnidaria showed high model misclassification rates with *A. labiata* and Scyphozoa. This result restates the challenge of distinguishing general cnidarian classifications at finer taxonomic levels. *P. bachei*, despite being a species-level taxon, was exclusively misclassified as background. Closer examination of test data suggests that this was likely due to limited training data and the fact that most instances of *P. bachei* appeared in high-turbidity conditions or at a distance. This finding is further supported by the observation that *P. bachei* was not confused with any other organism and had the lowest number of test images of any gelatinous zooplankton taxon (TP = 27). Similar challenges in classifying gelatinous zooplankton have been reported in other studies, where the morphological appearance (including translucent body) of gelatinous taxa may limit identification accuracy and performance (e.g., [38,39]).

For the gelatinous zooplankton and Cnidaria groupings, misclassification patterns were similar to those seen in fish pseudo-taxa, where the model had difficulty separating broader taxonomic classifications from the background or other general categories. Unknown gelatinous zooplankton were commonly misidentified as background (85 cases), while Cnidaria was most often confused with the broad unknown gelatinous zooplankton group (0.17). Similar to fish pseudo-taxa, this challenge is likely related to the lack of visually distinct morphological features in these groups, which aligns with issues faced by other studies monitoring gelatinous zooplankton diversity from pelagic imagery [38]. However, despite the difficulty in classification, accurately quantifying these broad pseudo-taxa is essential for studies that seek to accurately estimate abundance, as they represented the majority of gelatinous zooplankton detections in our dataset. As with fish pseudo-taxa, future studies seeking to estimate true abundance of gelatinous zooplankton species could benefit from standardizing the annotation effort and potential biases introduced by our algorithmic screening workflow.

### 4.2. Experiment 2: Model Evaluation at Multiple Annotation Thresholds

#### 4.2.1. Performance Differences Across Pseudo-Taxa

We retrained our final YOLOv8 model on a subset of annotated frames (58,544 annotations across 10 pseudo-taxa) from the initial project. Overall, mean detector performance (F1) and mean-average precision (mAP50) began low at the 25 annotation per group threshold (<0.35 and <0.2, respectively) and finished at 0.91 and 0.84, respectively, at the end of the experiment. The object detection model did not achieve an mAP50 or F1 ≥ 95% for any group in the dataset at any annotation threshold. Interestingly, neither metric appeared to reach an asymptote during the final model runs, suggesting that more annotations may have led to better model performance.

Our model performance aligns broadly with similar studies, while suggesting that future research could improve on our method through recent advancements in underwater object detection pipelines (e.g., [40,41]). For instance, Li et al. (2024) [41] introduced Underwater-YOLO, a modified YOLO-based model which achieved an average precision at 50% intersection over union (mAP50) of 93.8% on a range of fish taxa. However, unlike the present study, recent research has used algorithmic filters to improve image quality and account for variable light conditions during model training [40]. Another technique for underwater YOLO-based pipelines involves first optimizing the use of high-quality and diverse annotations in training a CV model (i.e., K-means clustering to derive anchor box distributions), further supporting the need for CV workflows specific to underwater imagery [41]. Similarly, research on the well-known Common Objects Underwater (COU) dataset demonstrated that increasing the number of annotated underwater images can considerably improve the performance of object detection models [42]. The COU dataset, comprised of images from a variety of underwater backgrounds, cameras, and recordings, resulted in better model performance and accuracy compared to models pretrained solely on terrestrial data such as COCO [42]. This finding demonstrates one of the key benefits of using contextually similar and robust underwater annotated datasets to train object detection models.

When examining model performance by individual group, some groups required fewer annotations than others to achieve high model performance. While *A. flavidus* and drift algae both achieved near −90% F1 performance and mAP50 within 500 annotations per class, some pseudo-taxa, like unidentified forage fish, Actinopterygii, and Scyphomedusae took several thousand annotations or never reached F1 performance over 0.9. As with the previous experiment, this discrepancy is likely due to the inherent morphological variability and low visual distinctiveness of these groups. The worst-performing pseudo-taxa in the present study were all from higher-level classifications (e.g., order, superfamily, or family), which has been found in other areas of ecological CV research. For instance, studies on the image classification of Amazonian parrots [24] and insects [43] have demonstrated that integrating taxonomic hierarchies into model training can improve accuracy, but including broader taxonomic groups can potentially lead to challenges in identification due to their within-class variability. Similarly, other recent underwater YOLO object detection studies have also found it challenging to detect small, dense, or occluded objects (common traits amongst generalized taxa) and suggest using custom feature extraction CV tools (e.g., post-processing steps) to improve performance for these types of groups [12,26,40].

These findings highlight the importance of high-quality annotations, model design, and dataset composition in CV underwater monitoring. In our study, more specific pseudo-taxa were easier to detect with fewer annotations, while those with greater visual variability, like unknown forage fish and other broad pseudo-taxonomic groups, required more training data. We believe that challenges such as light attenuation, occlusions, and small animal sizes likely impacted detection accuracy, but also that technological advancements (e.g., model architecture, image augmentation and other preprocessing techniques) have the potential to make these issues less impactful in the future. Future research should explore annotation strategies and classification techniques to directly address these common challenges, which would further improve underwater object detection benchmarking and support long-term CV-assisted ecological monitoring.

Future research could improve on our annotation approach by using more targeted selection methods to increase annotation efficiency (e.g., [15]). For instance, Mac Aodha et al. (2019) [44] proposed using geographical priors (i.e., the use of location-based information to guide model training and annotation strategies) to inform annotation in image classification for creating ecological training datasets, prioritizing more informative samples for labeling. As with highly variable pseudo-taxa, focusing annotation efforts on the most uncertain or ecologically relevant cases may increase model generalization and accuracy for underwater species identification [15,44].

#### 4.2.2. Shared Feature Learning Results

##### Effect of Shared Feature Learning on Low-Annotation Pseudo-Taxa

We saw continued improvement in model performance for pseudo-taxa with the fewest annotations, despite little or no increase in their annotations. This effect is likely due to shared learned upstream features, where models trained on additional data learn more generalizable features such as shape, color, and edge detection that can be applied across pseudo-taxa [45]. In our study, the YOLOv8 model may have benefited from common pseudo-taxa with high numbers of annotations, thereby allowing underrepresented groups to improve without respective increases in annotations from those taxa. This result aligns to other recent studies in this field, which show that convolutional neural networks (CNNs) trained on varied datasets (in background and diversity of animals) can improve classification accuracy on visually similar, but underrepresented, categories (e.g., [18,46]).

Salmonidae, *Sebastes* sp., and Ctenophora had the largest increase in F1 performance in subsequent training runs. One possible reason for this result is that these pseudo-taxa share visual features with better-represented groups, thereby improving model identification accuracy via exposure to similar object features. If accurate, this outcome means that the model achieved higher precision and recall for a test dataset for some pseudo-taxa simply by having more information of other visually similar (but different) groups. This phenomenon aligns with findings from other ecological CV-related studies, where models benefit from shared feature learning across other related taxa [36,43,46]. For instance, Kim et al. (2025) found that hierarchical computer vision models used in monitoring Amazonian parrots had higher identification accuracy among species that were morphologically similar [24]. This result suggests that shared feature learning by using groups of visually similar taxa can improve model performance, as in our study where pseudo-taxa with fewer annotations benefited from the model’s training on better-represented groups. However, given feature sharing or transfer is a well-documented phenomenon that occurs even with dissimilar training data (e.g., training first on large datasets like COCO are common, regardless of the desired objectives for the model), we expect that our model would have improved for annotation-deficient groups even if the training data were not visually similar as the low-level features intrinsic to images are still similar.

The continuous improvement of some pseudo-taxa in later model runs without additional training photos supports the benefit of cumulative model learning. The high negative correlation (R = −0.97) between previous performance and relative improvement in later runs supports this finding, since pseudo-taxa that initially performed badly improved more. Since pseudo-taxa with great starting accuracy had little space for improvement, this pattern was expected. For example, drift algae declined by 0.3% in later model runs, which we expect is due to a combination of the model’s high initial performance for the group, coupled with within-run error. While research in ecological shared feature learning is limited, this result aligns with a study in plant taxa where CV model refinement through repeated training cycles lead to considerable improvements in low-performing classes [47]. We recommend that future studies in similar underwater systems iteratively monitor model performance across taxa, and implement strategies to balance the representation of groups based on model performance.

## 5. Conclusions

This study assessed the performance of a YOLOv8 object detection model on fish and gelatinous zooplankton pseudo-taxa, with a focus on trends in classification performance and misclassification patterns. The model tended to perform well on species or genus-level pseudo-taxa with distinct morphological features, but misclassification rates were greater for larger pseudo-taxonomic groups and those with considerable intra-class diversity, such as unidentified forage fish, Actinopterygii, and Cnidaria. These errors were frequently systematic in nature, with the model usually confusing visually similar pseudo-taxa with one another or the background, which anecdotally matches the challenges annotators experienced with these groups (i.e., annotators found general groups of taxa more challenging to assign labels to). However, our results underscore the challenges and importance of incorporating general fish and gelatinous zooplankton pseudo-taxa in monitoring efforts, as we show (through annotation data collected) that omitting these groups would lead to severe underestimations of overall fish abundance.

In re-training the YOLOv8 model on a subset of our data, we found that the number of training annotations played a crucial role in model performance, but the benefit of additional annotations varied by pseudo-taxa. While some groups, such as drift algae, achieved high accuracy with relatively few annotations, others, including unidentified forage fish and Scyphomedusae, required extensive training data and still exhibited lower performance. Our study also demonstrated that shared model training improved detection accuracy for low-annotation pseudo-taxa, likely due to feature sharing from better-represented groups. However, this benefit was inversely proportional to previous model performance for these groups, suggesting that shared feature learning may be best implemented to boost performance in rare pseudo-taxa with features that are similar to more common groups, which may not be recognized well by CV models. These results highlight the importance of taxon-specific annotation strategies. Future research should explore targeted annotation methods for specific pseudo-taxa of interest and model performance based on broad versus specific (e.g., species-level) pseudo-taxa and build upon the detector development and video validation methods presented in this study.

## Figures and Tables

**Table 1:** Summary of annotations made for fish pseudo-taxon categories that comprised the FishCam training dataset. The “schooling” and “exemplar” labels were unique to schooling fish pseudo-taxa; groups with an “exemplar” label indicate the number of annotated individuals that were in focus and used to help identify other fish in a school, whereas a “schooling” label was applied to individuals within a pseudo-taxon that could not be identified based on their features alone, but rather by the presence of an “exemplar” individual in the school.

Pseudo-Taxon Group	Common Name	Number of Annotations Per Category	Total Annotations Across Related Categories
Actinopterygii	Unknown Fish	5997	5997
Unidentified forage fish	Unidentified forage fish	15,189	15,189
*Aulorhynchus flavidus*	Tubesnout	448	488
*Aulorhynchus flavidus (exemplar)*	Tubesnout	26	
*Aulorhynchus flavidus (schooling)*	Tubesnout	14	
Salmonidae	Unknown salmonid	114	214
*Oncorhynchus tshawytscha*	Chinook salmon	1	
*Oncorhynchus tshawytscha or Oncorhynchus kisutch*	Chinook or coho salmon	25	
*Oncorhynchus tshawytscha or Oncorhynchus kisutch* (juvenile)	Chinook or coho salmon (juvenile)	32	
Unknown juvenile salmonid	Unknown juvenile salmonid	42	
*Sebastes* sp.	Unknown rockfish species	686	809
Banded rockfish complex	Banded rockfish complex	2	
*Sebastes flavidus*	Yellowtail rockfish	64	
*Sebastes flavidus or Sebastes melanops*	Yellowtail or black rockfish	57	
Clupeiformes (exemplar)	Herrings	29	3507
Clupeiformes (schooling)	Herrings, schooling	3478	
*Clupea pallasii* (exemplar)	Pacific Herring	347	6004
*Clupea pallasii* (schooling)	Pacific Herring	5657	
*Engraulis mordax* (exemplar)	Northern anchovy	65	716
*Engraulis mordax* (schooling)	Northern anchovy	651	
Embiotocidae	Surfperches	7128	8772
Embiotocidae (exemplar)	Surfperches	812	
Embiotocidae (schooling)	Surfperches	832	
*Cymatogaster aggregata*	Shiner perch	4809	6178
*Cymatogaster aggregata* (exemplar)	Shiner perch	234	
*Cymatogaster aggregata* (schooling)	Shiner perch	357	
*Cymatogaster aggregata* (dark morph)	Shiner perch—male breeding color	778	
*Phanerodon vacca* (exemplar)	Pile surfperch	152	1693
*Phanerodon vacca* (schooling)	Pile surfperch	54	
*Phanerodon vacca*	Pile surfperch	1487	
*Brachyistius frenatus*	Kelp perch	25	25
*Embiotoca lateralis*	Striped surfperch	35	35
Total annotated fish			49,627

**Table 2:** Summary of annotations made for gelatinous zooplankton categories that comprised the FishCam training dataset.

Pseudo-Taxon Group	Common Name	Annotations Per Category	Total Annotations Across Related Categories
Pelagic Cnidarian Medusae and Ctenophores	Gelatinous zooplankton	5671	19,835
Cnidaria	Jellyfishes	605	12,860
Hydromedusae	Hydromedusa jellyfishes	2240	11,622
Visually similar group: Hydromedusae Jellyfishes	Cross jelly complex	184	
*Neoturris breviconis*	Blob-top jellyfish	33	
*Sarsia* sp.	Thimble jellyfishes	6	
*Praya* sp.	Giant siphonophore	130	
Melicertidae	Eight-strand jellyfishes	11	
*Polyorchis* sp.	Red-eye medusa jellyfishes	89	
*Aequorea* sp.	Water jellyfishes	8751	
*Eutonina* sp.	Aggregating jellyfishes	33	
Ctenophora	Ctenophore jellies	96	595
Lobata	Comb jellies	71	
*Beroe* sp.	Cigar comb jellies	10	
*Cydippida*	Lobed comb jellies	57	
*Pleurobrachia bachei*	Sea gooseberry	257	
Bolinopsidae	Orange-tipped sea gooseberry	104	
Scyphomedusae	True jellyfishes	218	1238
*Aurelia labiata*	Moon jellyfish	712	
Visually similar group: *Cyanea capillata or Phacellophora camtschatica*	Fried Egg Jellyfish or Lion’s Mane Jellyfish	294	
*Phacellophora camtschatica*	Fried Egg Jellyfish	14	
Total annotated gelatinous zooplankton			19,835

**Table 3:** Total number of annotations and relative proportion for each pseudo-taxon or taxon in the dataset.

Taxon Group	Common Name	Annotations in Dataset	Proportion
Forage fish	Forage fish	23,346	40.81%
Embiotocidae	Surfperches	13,941	24.37%
Hydromedusae	Hydromedusa jellyfishes	10,188	17.81%
Actinopterygii	Unidentified bony fishes	5310	9.28%
Scyphomedusae	True jellyfishes	1980	3.46%
*Sebastes* sp.	Rockfishes	765	1.34%
Ctenophora	Ctenophore jellies	657	1.15%
Drift algae	Drifting algae	423	0.74%
*Aulorhynchus flavidus*	Tubesnout	414	0.72%
Salmonidae	Salmonids	180	0.31%

**Table 4:** Precision, recall, mean average precision of the 50th percentile (mAP50), and mean average precision between the 50th and 95th percentiles (mAP50-95) for pseudo-taxa included in the YOLOv8 object detection model. Annotations represent the number of images and annotations used for each taxon in the test dataset, respectively. There were 8376 images total in the test dataset. Of the 54 pseudo-taxa identified during annotation, groups with under 200 annotations were aggregated to their respective parent taxa, yielding 23 pseudo-taxa used to train the final model.

Class	Annotations	Precision	Recall	mAP50	mAP50-95	F1
All groups	7692	0.705	0.784	0.784	0.536	0.742
Actinopterygii	621	0.543	0.678	0.644	0.415	0.603
Embiotocidae	743	0.668	0.849	0.839	0.599	0.748
*P. vacca*	148	0.807	0.959	0.944	0.726	0.876
*Aequorea* sp.	884	0.868	0.948	0.951	0.673	0.906
*C. aggregata*	489	0.842	0.933	0.93	0.679	0.885
Cnidaria	60	0.573	0.633	0.679	0.455	0.602
*A. labiata*	69	0.896	0.913	0.955	0.687	0.904
Gelatinous zooplankton	488	0.643	0.748	0.746	0.457	0.692
Drift algae	53	0.914	0.906	0.936	0.649	0.910
*Sebastes* sp.	78	0.849	0.94	0.956	0.676	0.892
*A. flavidus*	49	0.88	0.918	0.947	0.631	0.899
Scyphozoa	98	0.656	0.724	0.71	0.409	0.688
*P. bachei*	33	0.799	0.846	0.868	0.479	0.822
Hydromedusae	250	0.638	0.775	0.786	0.547	0.700
*C. aggregata* (dark morph)	83	0.797	0.952	0.91	0.676	0.868
Scyphomedusae	19	0.657	0.947	0.844	0.584	0.776
Embiotocidae (exemplar)	98	0.618	0.745	0.706	0.456	0.676
Unknown forage fish	1887	0.535	0.746	0.598	0.318	0.623
*C. aggregata* (exemplar)	26	0.54	0.462	0.477	0.371	0.498
*C. capillata* or *P. camstchatica*	33	0.838	0.939	0.966	0.712	0.886
Ctenophora	29	0.734	0.966	0.942	0.621	0.834
*C. pallasii* (exemplar)	56	0.524	0.696	0.637	0.413	0.598
Salmonidae	29	0.798	0.966	0.92	0.743	0.874

## Data Availability

The data presented in this study will be made available upon reasonable request to the corresponding author; restrictions may apply as some data are part of an ongoing study.

## Supplementary Materials

The following supporting information can be downloaded at: https://www.mdpi.com/article/10.3390/s26185869/s1, Figure S1: Hierarchical tree for all taxonomic and visually-similar groups in the image annotation dataset. Solid lines indicate taxonomic groups, and dotted lines indicate visually similar (pseudo-taxa) groups. Epiphytic animals (e.g., Caprellidae, Tunicata) were not included for the purposes of this project.
