# Supplementary material for: Underwater Computer Vision for Ecologists: A Framework for Curating Image Training Datasets for Object Detection
Source: Sensors (Basel). 2026 Sep 16;26(18):5869. doi: 10.3390/s26185869 (PMC13611200; doi:10.3390/s26185869)
Supplement: Supplementary file 1 [file sensors-26-05869-s001.zip › sensors-4421083-supplementary.pdf]

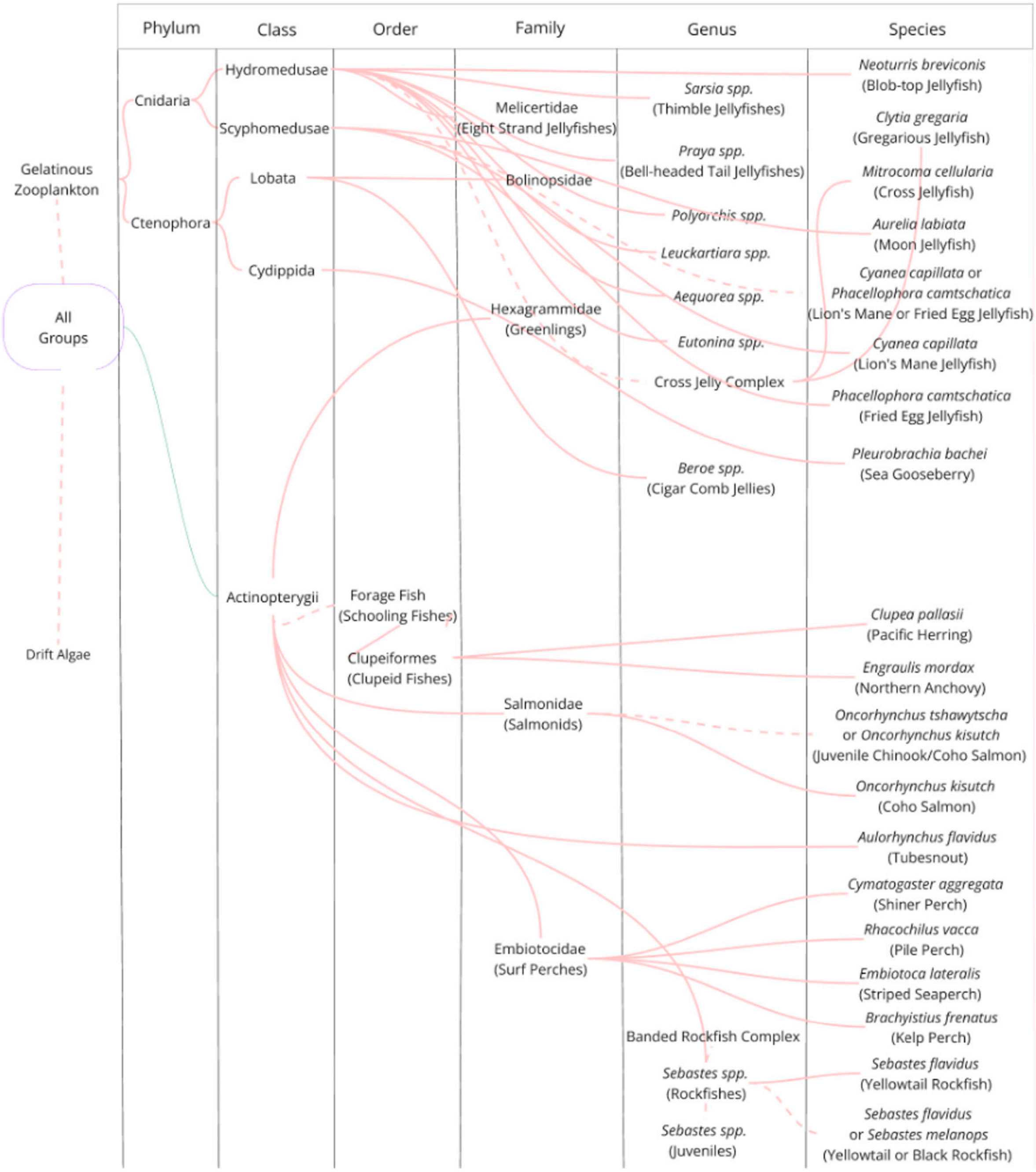

Figure S1: Hierarchical tree for all taxonomic and visually-similar groups in the image annotation dataset. Solid lines indicate taxonomic groups, and dotted lines indicate visually similar (pseudo-taxa) groups. Epiphytic animals (e.g. Caprellidae, Tunicata) were not included for the purposes of this project
